# Self-isolated MIMO antenna using mixed coupling by close coupling technique

**DOI:** 10.1038/s41598-023-32364-3

**Published:** 2023-04-06

**Authors:** Harsh Verdhan Singh, D. Venkata Siva Prasad, Shrivishal Tripathi

**Affiliations:** Electronics and Communication Department, International Institue of Information Technology Naya Raipur, Naya Raipur, 493661 India

**Keywords:** Electrical and electronic engineering, Electronics, photonics and device physics

## Abstract

A self-isolated multiple-input-multiple-output (MIMO) antenna in a compact shared ground structure is proposed for 5G systems. The proposed MIMO antenna consists of customized M-pattern, closely coupled members. It benefits to attain good isolation of the targeted bandwidth transversely without additional de-coupling structures. It is discovered that the arm of M-pattern antenna members can cancel out the coupling on the system and achieve sound isolation among antenna members. A relevant matching circuit model is discussed to show how the suggested theory works in principle. The mixed couplings among antenna members neutralize by modifying the antenna shape with the help of electric and magnetic coupling and surface currents. The proposed self-isolated 2-member MIMO antenna demonstrates sound isolation superior to 15 dB transversely in the frequency bands dedicated to 5G NR: n48/n78 and long-term evolution (LTE) band 42/43/48/52 (3.2–3.98 GHz). Moreover, the proposed 2-member design structure has a scalability advantage. It is extended into an 8-members structure, where a pair of antennas located at each side of the frame offers orthogonality. The proposed 8-members M-pattern MIMO is validated using fabricated and simulated measurements. The investigational results show that the 8-members MIMO (M-shaped) system is effective in higher order MIMO antenna design and offers more than 20 dB isolation transversely in the frequency band 5G NR n48 and LTE band 42/43/52(3.29–3.66 GHz).

## Introduction

MIMO technology is extensively employed in wireless communication systems to address the growing need for better service quality, lower latency, and higher data rates. Several antennas lay on in the system have encountered new issues in fulfilling the overhead demands^1,2^. Particular of the concerning issues is the short isolation among the closely coupled antennas that are utilized in a compact device, which weakens the MIMO systems performance^3^. So, it is precarious and essential to discover self-deprecating and dynamic isolation methods for the MIMO system.

Numerous approaches in the previous works of MIMO antennas to increase the isolation have neutralization line, defected ground structure (DGS), metamaterial, and Electromagnetic Band Gap (EBG)^3–14^. The narrow open-ended slots and space diversity^3^, DGS in the ground plane^4^^,^^7^, the vertical ground strip with EBG structure in the top plane orthogonally arranged patch antenna^5^, the shorting stub^6^, the complementary-split‐ring-array (CSRA)^8^, loading split-ring-resonators (SRR)^9–11^, complementary slot coupled SRR^12^, the planar EBG structure^13^, 2nd order de-coupling circuit^14^ are applied in the MIMO antenna, which in turn leads to isolation improvement. Phase-gradient metasurfaces (PGMs) are used to deflect an antenna beam in a preferred direction^15^. The hybrid dispersion-engineered metamirrors for dispersion manipulation of the antenna are utilized^16^. Nonetheless, most overhead works increase isolation and change the antenna properties with the help of supplementary design, which could change the characteristics of the antenna. Nevertheless, these techniques upsurge the intricacy of the design pattern with a rising number of members. Although the techniques revealed above show appealing traits, they experience issues and complexities generated with the aid of supplementary systems.

Various self-isolation-based approaches are offered in literature^17–25^. There are techniques that works with common coupled grounding branch^17^, common-mode(CM) and differential-mode(DM) tunning^18^, using ground plane weak-field^19^, gap-coupled loop antennas using asymmetrically mirrored arrangement^20^, L-pattern branch operates as a radiating and de-coupling element^21^, inverted U-pattern member^22^, using closely paired^23^, couplings between a pair of coupled antennas are canceled out by proper adjustment^24,25^. The shorting legs in the PIFA pair^6^ are utilized to attain self-isolation in the MIMO antenna. However, one of the above works primarily employs an opposed location to de-couple the antenna members. Moreover, one paper offered a weak field concept, and one more displayed signaling to tune using CM and DM concepts to de-couple the antenna members. Looking at the self-isolation MIMO antenna system's ostensible benefits is necessary.

In this work, a self-isolated technique is projected for the MIMO antenna structure. The offered works have the following contributions related to the other MIMO antenna.

1. The mixed-coupling effect using the close coupling technique is introduced and managed without additional de-coupling circuits with the neighboring members. The magnetic and electric coupling combination agreements surface current cancellation and provide self-isolation.
2. The central concept is to equilibrium the coupling between the antenna members employing stretched arms adjusting mixed-coupling among the elements.
3. The presented structure is compressed, likened to earlier papers because the system's tightly coupling designed structure attains the self-isolation result. The monopoles of the antenna are placed close together to offer sufficient capacitive and inductive coupling to the other element.
4. The proposed MIMO antenna members are easily extended to more than a 2-member array due to the compactness and easily integrable of the proposed design structure.
5. The flexible integrability of waveguide and coaxial feed is achieved easily due to compactness and self-isolation scenarios as presented in the design.
6. The MIMO antenna self-isolation characteristics deal with isolations better than 16 dB for the 2-members structure and 20 dB for the 8-members design throughout the operating band. The shortest distance among antenna members is about 0.2 mm (0.005λ_g_ at 3.8 GHz).

The projected 2-member MIMO (M-shaped) antenna can offer the 3.5 GHz band (3.2–3.98 GHz), and the 8-member MIMO (M-shaped) antenna can cover the 3.5 GHz band (3.29–3.66 GHz), which is directed for the approaching 5G band for sub-6 GHz range. MIMO antenna pair with a common ground to develop MIMO antenna integration levels and house the upcoming 5G systems. The mobile frame-based eight members MIMO antenna example is presented with user hand effect analysis.

### 2-Member MIMO (M-shaped) antenna design

#### Antenna prototyping

**Figure 1:**
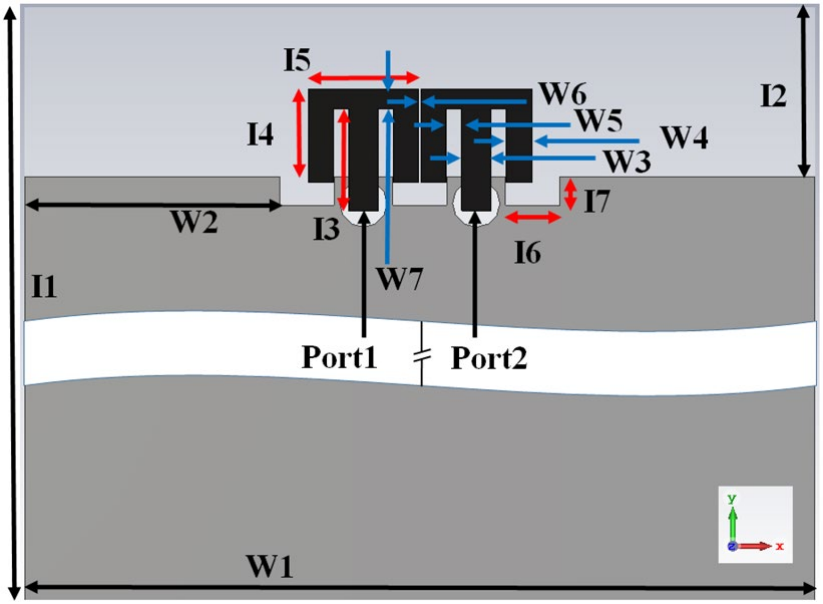
Optimized design of closely-couple 2-Member MIMO (M-shaped) antenna structure.

**Figure 2:**
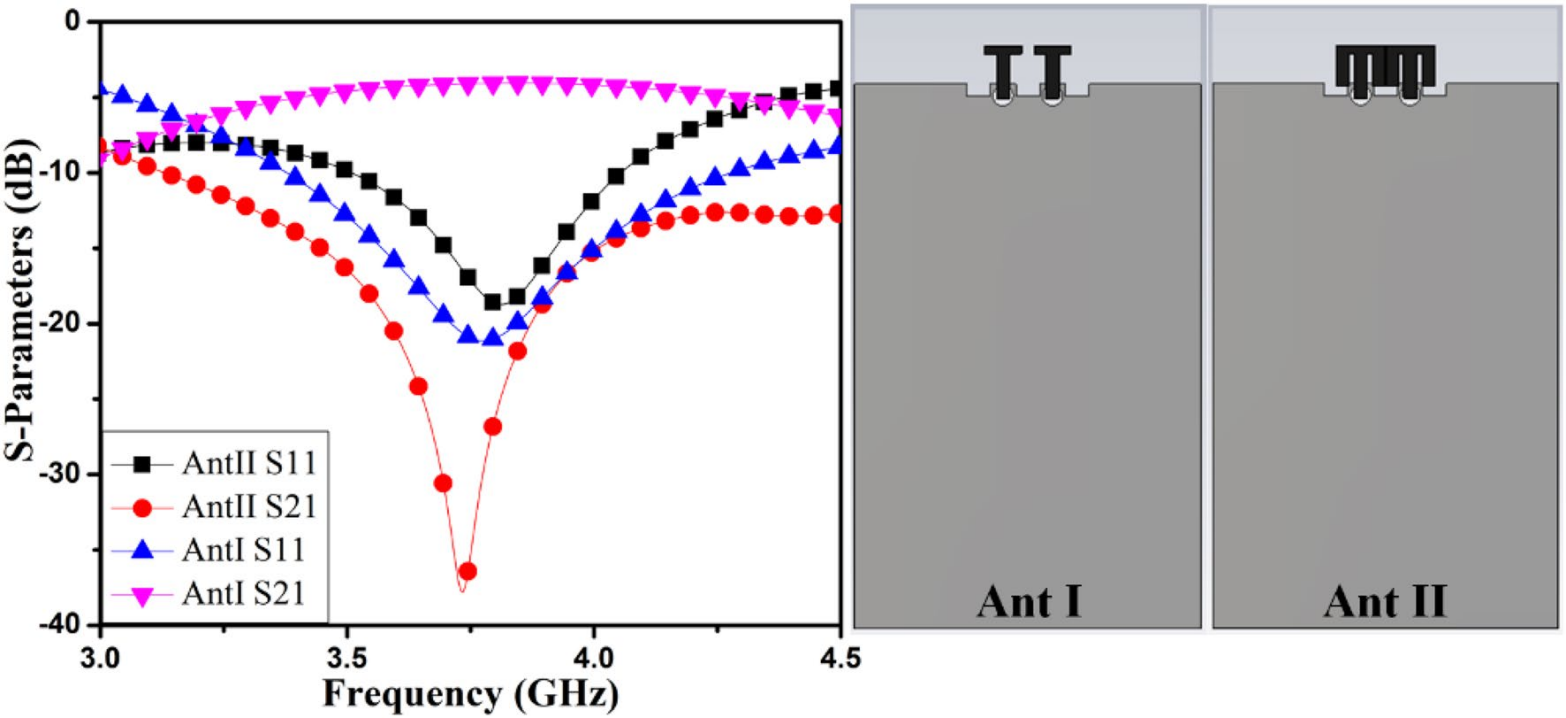
Evolution of MIMO antenna S-parameters.

The self-isolated closely coupled MIMO (M-shaped) antenna member is represented in Fig. 1. The closely associated customized M-pattern radiating member is used in this projected configuration. The tightly placed antenna elements can provide good capacitive and inductive coupling to the other element; tuning the present coupling helps achieve self-isolation. It provides self-isolation characteristics without using any de-coupling circuit. The system measurements are 125 mm × 70 mm × 1.6 mm, whereas the ground measurement is 110 mm × 70 mm. Dual compact antenna members are configured and positioned at the upper sections of the frame. It is located in the center position of the frame. The presented antenna comprises dual same customized M-pattern antenna members. Its evolution (AntI and AntII) is shown in the inset of Fig. 2, with S-parameters simulated results.

**Figure 3:**
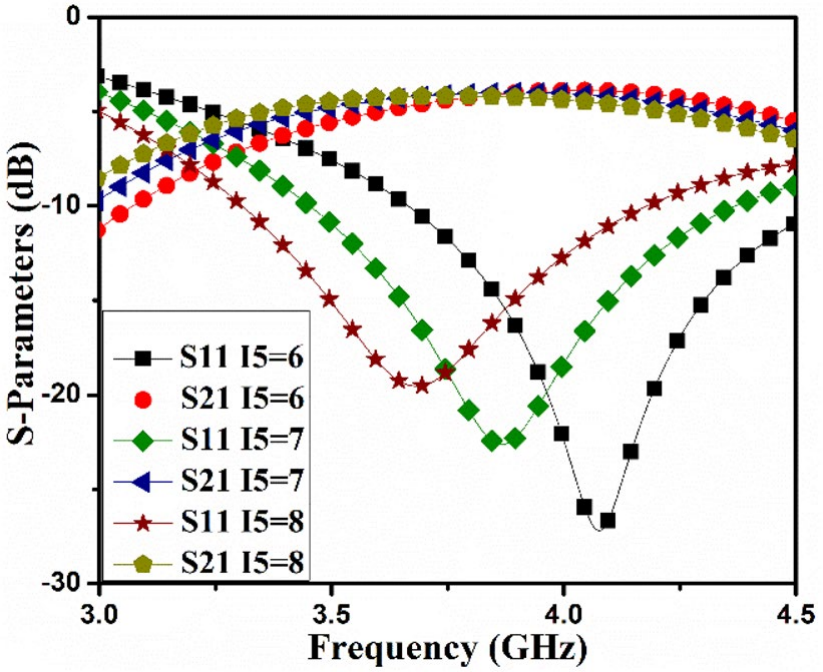
AntI S-parameter varies as a function of I5.

In the initial configuration, a T-pattern member is employed in the MIMO antenna projected as AntI, as shown in the inset of Fig. 2. The AntI members are positioned next to one another, and about *4 dB* isolation of coupled antenna members offered at the center frequency. The length *I5* of the T-pattern configuration adjusts the center frequency, as presented in Fig. 3.

In the next evolution, the T-pattern structure is customized as AntII, as shown in the inset of Fig. 2. In this customized structure, two arms are incorporated in a T-pattern structure. The customized M-pattern monopole structure is closely coupled with another monopole. The inter-member spacing among antenna members is around *0.2 mm* (*0.005λ*_*g*_ at *3.8 GHz)*. The customized arm of the presented structure provides sound isolation among the antenna members with the help of ground cuts *I6,* and *I7*.

The passive matching circuit is used to comprehensively analyze the projected self-isolated MIMO antenna. The MIMO antenna suffers coupling owing to capacitive and inductive influences between the members. The inductive and capacitive effects occur due to the adjustment of the structure. The maximum electric field between the members offers capacitive coupling, and magnetic fields at the edge of the customized design offer inductive coupling. This effect can be manipulated deliberately and abolish the coupling between the pair of members with the same magnitude with the help of surface current. The close-coupled arm of the antenna member produces surface currents to the equilibrium of the coupling introduced by the nearby antenna member. The following section describes the self-isolated designed MIMO (M-shaped) antenna matching passive circuit to determine the appropriate configuration.

#### Self-isolated MIMO (M-shaped) antenna analysis

**Figure 4:**
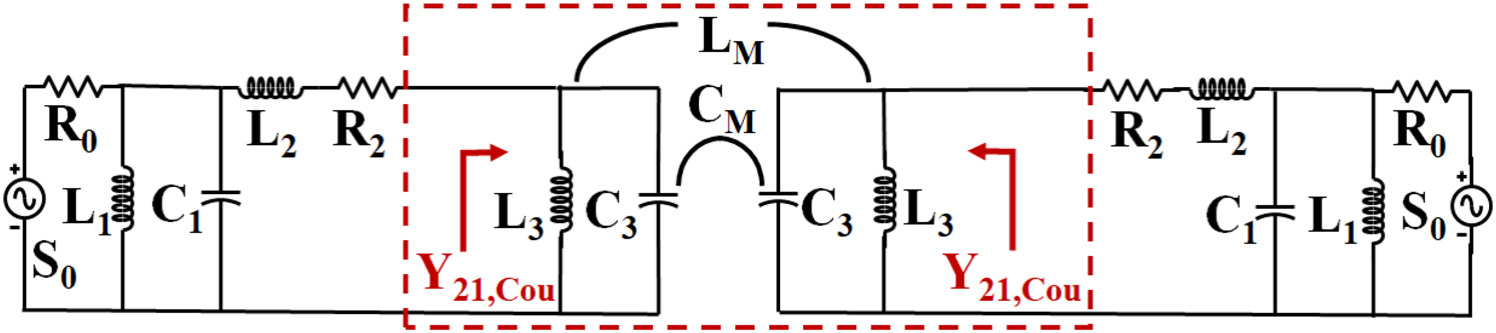
Matching passive circuit configuration of the MIMO (M-shaped) antenna.

The matching passive circuit is used to explain the concept of the projected MIMO antenna's structure, presented in Fig. 4. The matching circuit of antenna members is extracted by examination of the electromagnetic model and classification of circuit members^26–28^. The antenna members are represented by members *L*_*1*_*, C*_*1*_*, L*_*2*_*,* and *R*_*2*_. The coupling among the antenna members is presented inside the dotted red box as mutual admittance *Y*_*21,Cou*_, consisting of *L*_*3*_*, C*_*3*_*, L*_*M*_*,* and *C*_*M*_. The coupled antenna members consist of two coupling types (inductive and capacitive). The mixed inductive and capacitive coupling is represented in parallel circuit members. To cancel these couplings, conscious tuning is required. The coupling among antenna members is defined stated as *C*_*M*_ and *L*_*M*_ for capacitive and inductive coupling matching. In this instance, the mutual couplings *C*_*M*_ and *L*_*M*_ are capacitors and inductors among antenna members. The impact of the coupling influence can be analyzed using an enclosed circuit inside the red box. The mutual coupling influence of the proposed MIMO (M-shaped) antenna is confined in Fig. 4, using a red rectangle. Towards enhancing the designed antenna members' isolation, Y_21,Cou_ the coupling admittance needs to be modified; this helps to strengthen *Y*_*21*_ total mutual admittance. The *Y*_*21,Cou*_ can be signified as:

1 $${Y}_{21,Cou}=\frac{j}{\omega {L}_{M}(1-{{L}_{M}}/{{L}_{3}})}-j\omega {C}_{M}$$

**Figure 5:**
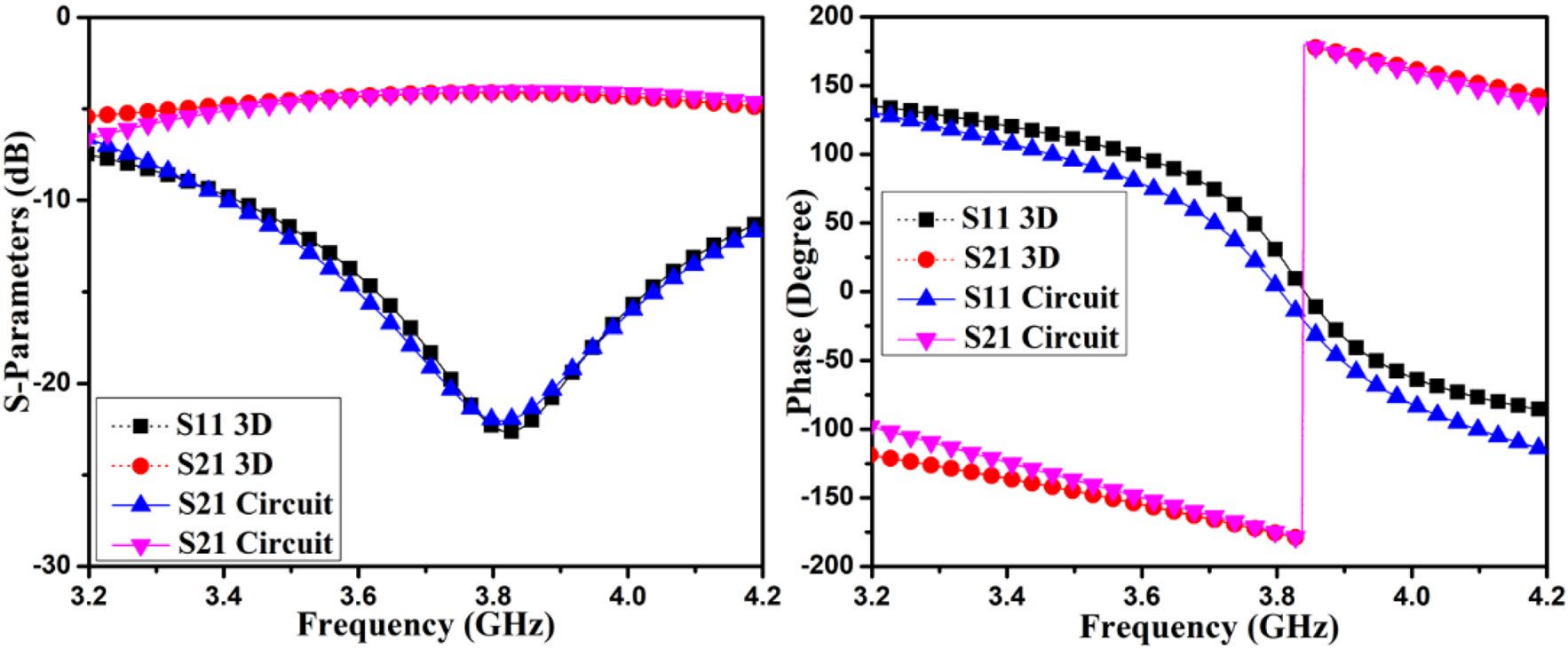
Phase plot and S-parameter simulated results of the 3D model and matching circuit of AntI.

**Figure 6:**
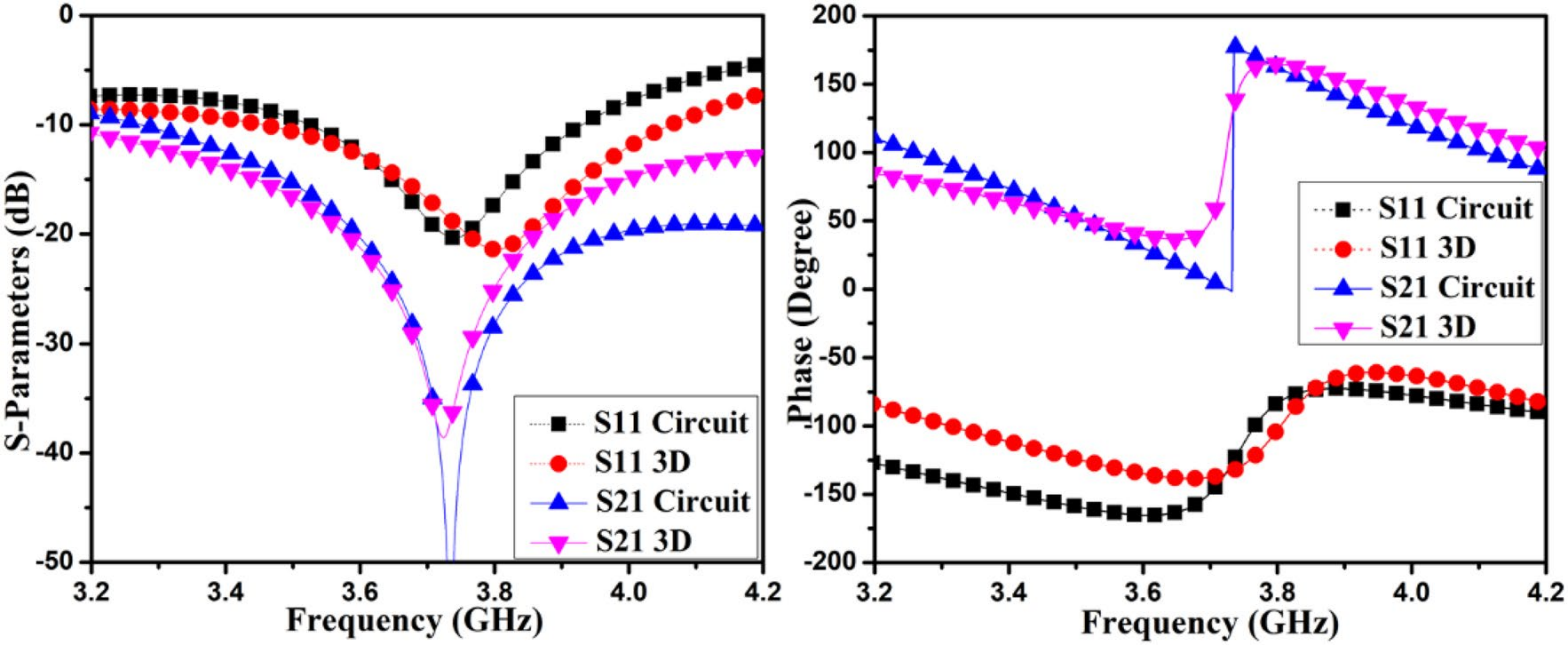
Phase plot and S-parameter simulated results of the 3D model and matching circuit of AntII.

Here ω represents radian frequency. The parameters *L*_*3*_*, C*_*M*_, and *L*_*M*_ adjusting can cancel out *Y*_*21,Cou*_ at a particular frequency. The circuit component values are estimated with the help of the Keysight ADS Simulator, and circuit values are listed in Table 1. The *R*_*2*_ are Ω, *L*_*1*_*, L*_*2*_*, L*_*3*_*, L*_*M*_, are in nH, *C*_*1*_*, C*_*3*_*, C*_*M*_, are in pF, and *Y*_*21,Cou*_ in mho. The *Y*_*21,Cou*_ mutual admittance at the middle frequency of AntI, and AntII is *-j4.057* and *–j0.908*, respectively. This displays good cancellation between the self-isolated MIMO antenna. The projected MIMO antenna 3D model and the phase plots and S-parameters simulated results of the matching passive circuit are shown in Figs. 5 and 6. The matching passive circuit and 3D model's S-parameters' phase and magnitude fit together quite well. The self-isolated MIMO (M-shaped) antenna concept is demonstrated with the help of the matching passive circuit.

**Table 1:** Matching passives element values for AntI and AntII.

	L_1_	C_1_	L_2_	R_2_	L_3_	L_M_	C_3_	C_M_
AntI	5.65	2.33	0.83	1.47	1.25	0.018	3.63	0.74
AntII	5.71	2.15	1.49	4.99	2.38	0.46	3.13	3.87

**Figure 7:**
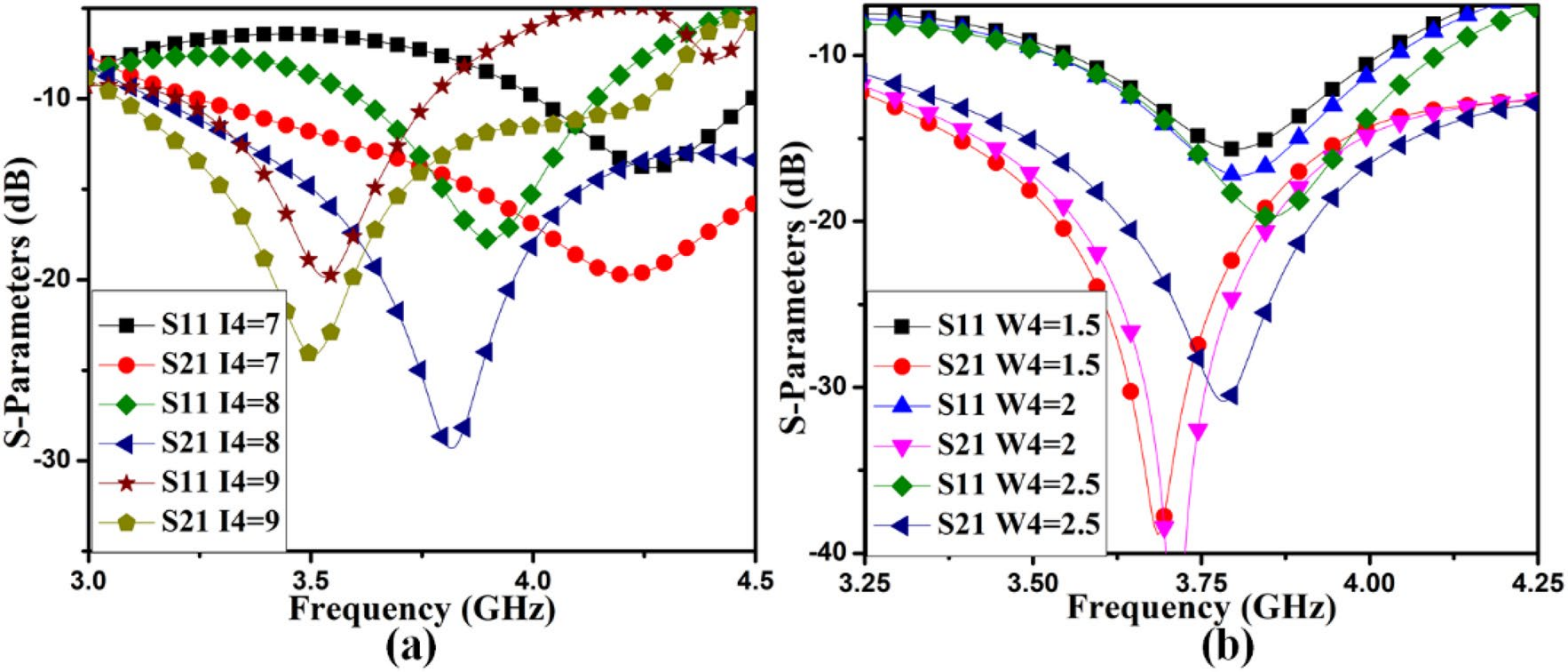
AntII S-parameter variation due to (**a**) I4, (**b**) W4.

**Figure 8:**
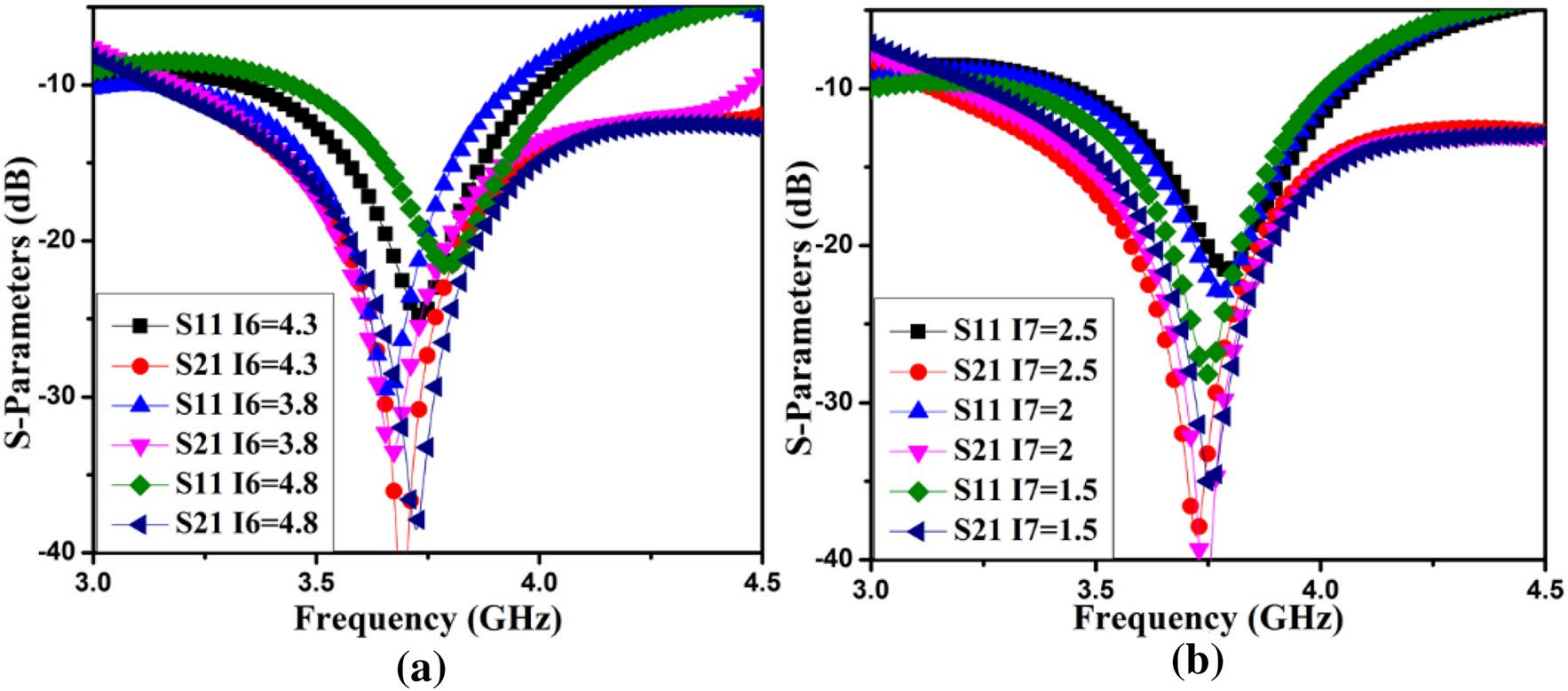
The S-parameter of the AntII operates with (**a**) I6, (**b**) I7.

It is realized from Fig. 7(a) that the *I4* differs from the self-isolated MIMO (M-shaped) antenna's central frequency. It is explicable because *I4* is the size of the radiating arm of the AntII. It is also noticeable that the isolation of the members is also tuned with it as well. The arm's width also impacts the S-parameters of the MIMO (M-shaped) antenna described in Fig. 7(b). The width is changed by keeping the edge-to-edge distance among the antenna member. So, arm’s width parametric variation occurs inside the structure of the arm. The width helps to tune isolation among the members. The ground alteration as *I6* and *I7* also helps to adjust the isolation of the MIMO antenna. The parametric variation due to *I6* and *I7* are illustrated in Fig. 8. It also provides small but significant variation in the isolation improvement.

**Figure 9:**
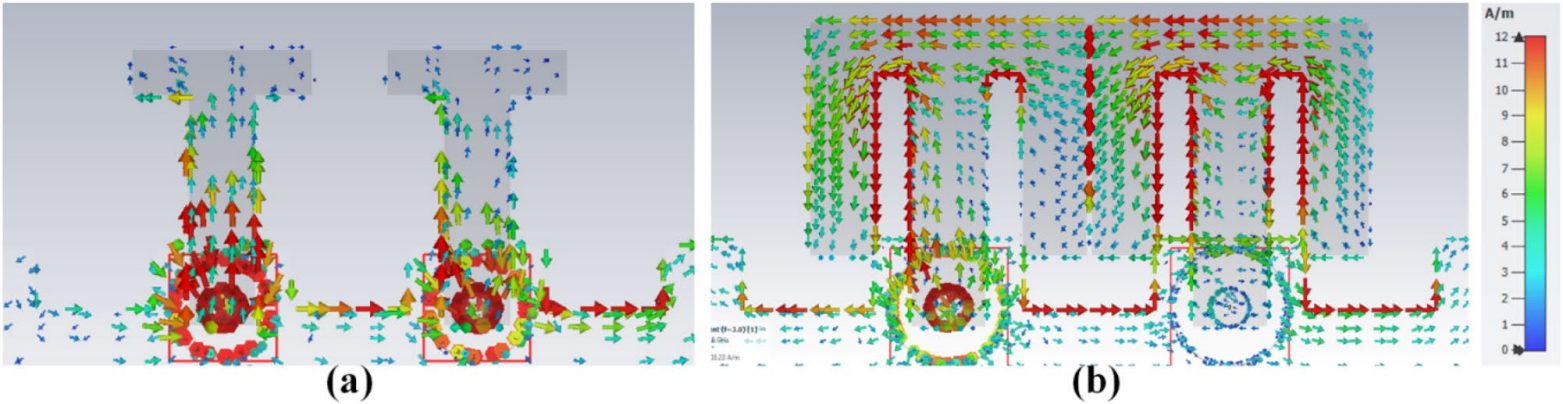
The density of surface current (**a**) AntI, (**b**) AntII at 3.8 GHz.

The customized M-pattern MIMO system performance can be improved by appropriately choosing the *I4*, *W4*, *I5*, and *I3* dimensions. The closely coupled arms of the MIMO members help improve the structure's isolation by coupled surface current neutralization, as demonstrated in Fig. 9. The electric field between the close coupled arm offers capacitive coupling. The circulating current from the leg to the arm offers inductive coupling to compensate for the coupling provided by the nearby structure. The combines capacitive and inductive coupling is mixed-coupling among the antenna pair and offers good isolation.

The projected customized M-pattern MIMO antenna radiates at 3.8 GHz with more than 25 dB isolation. The introduced MIMO antenna radiates from 3.5-4 GHz with an isolation of more than 15 dB. After the evolution of the customized MIMO (M-shaped) antenna, the improved dimensions are shown in Fig. 1. Measurements are made, and the results are shown in Table 2.

**Table 2:** Comprehensive measurement of projected MIMO antenna (units in mm).

I1 = 125	I3 = 9	I4 = 8.25	I7 = 2.5	I9 = 26	I11 = 5.9
W1 = 70	W3 = 2.6	W5 = 1.35	W7 = 1.75	W9 = 16	W11 = 2
I2 = 15	I5 = 9.8	I6 = 4.8	I8 = 140	I10 = 5.9	I12 = 5.9
W2 = 22.6	W6 = 0.2	I16 = 7.75	W8 = 70	I11 = 2.6	W16 = 2
W4 = 2.25	I13 = 5	W13 = 1.25	W14 = 2.6	W15 = 13	
I15 = 24.5	I14 = 8	W12 = 2.25	I17 = 4.8	W17 = 1.4	

Although the antenna members are tightly connected, this mixed coupled procedure enables the self-isolated characteristic of the offered MIMO antennas. An 8-member MIMO (M-shaped) antenna system is additionally validated for 5G systems, where the experimental results authenticate the viability of the projected technique.

### 8-Member MIMO (M-shaped) antenna example

As discussed in the last segment, the 2-member MIMO antenna presents self-isolating properties with excellent isolation in compact dimension isolation using mixed-coupling. These antenna member pairs can be extended to each side of the frame using a similar structure or the customized design shown in this section.

**Figure 10:**
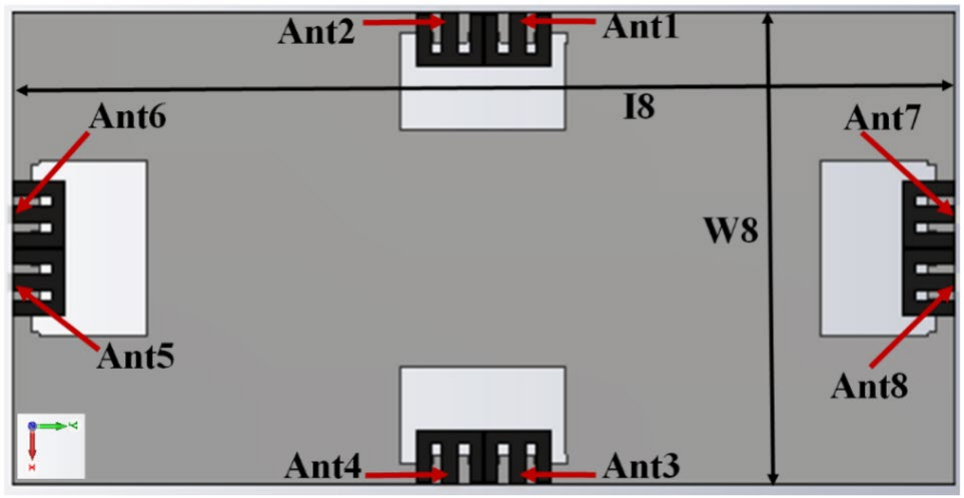
Optimized design of closely-couple 8-members MIMO antenna.

The closely coupled compact self-isolated 8-member MIMO antenna member is described in Fig. 10 for the upcoming 5G applications. In this projected configuration, the closely coupled M-pattern radiating structure is used. It provides self-isolation characteristics without using any de-coupling circuit. The dimensions of the system substrate are *140 mm* × *70 mm* × *1.6 mm*, while the ground size is similar to the dimension of the system with defects. A pair of closely coupled MIMO antennas are designed in the proposed system located at each side of the frame. The pair's orthogonality can help to improve the isolation among the other pairs of self-isolated MIMO antennas.

**Figure 11:**
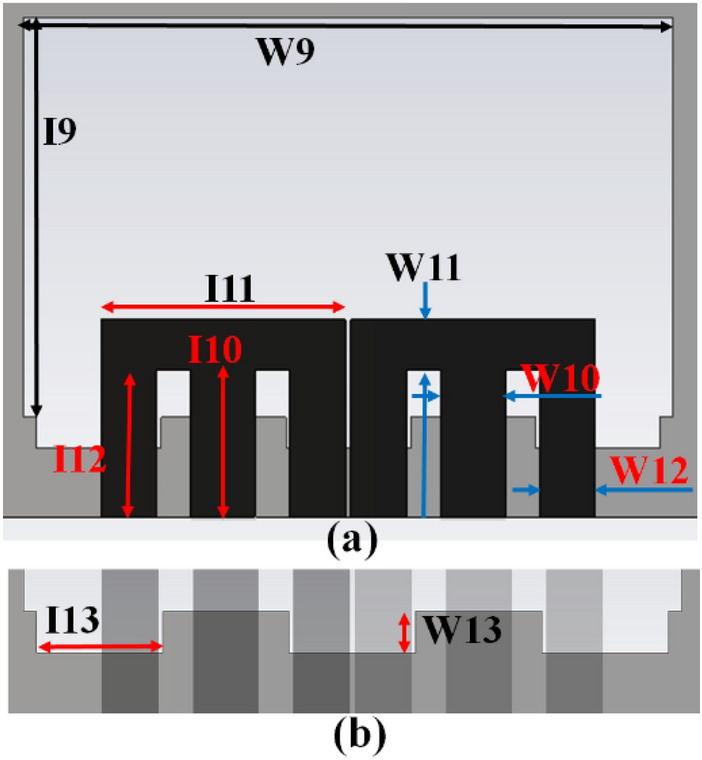
Detail dimension of Ant 5–8 (**a**) top, (**b**) ground structure.

**Figure 12:**
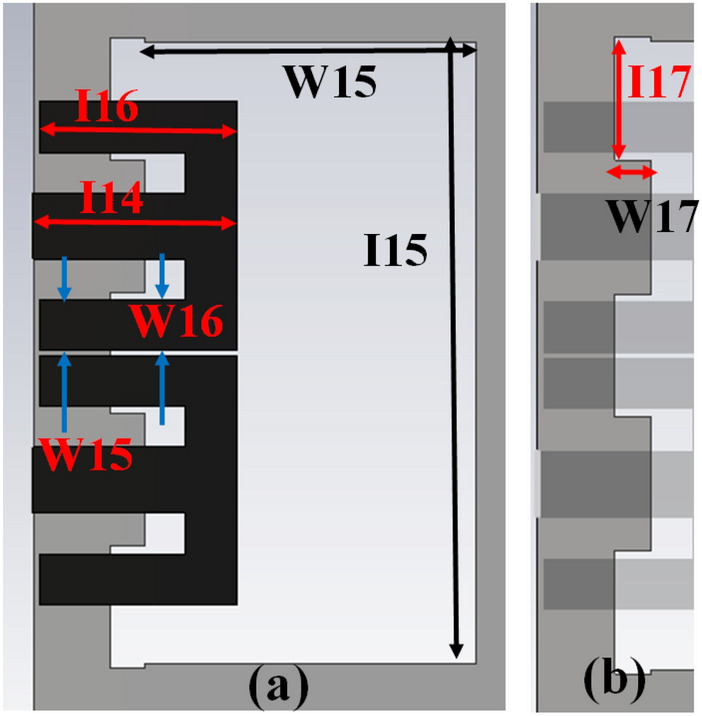
Detail dimensions of Ant 1–4 (**a**) top, and (**e**) ground structure.

**Figure 13:**
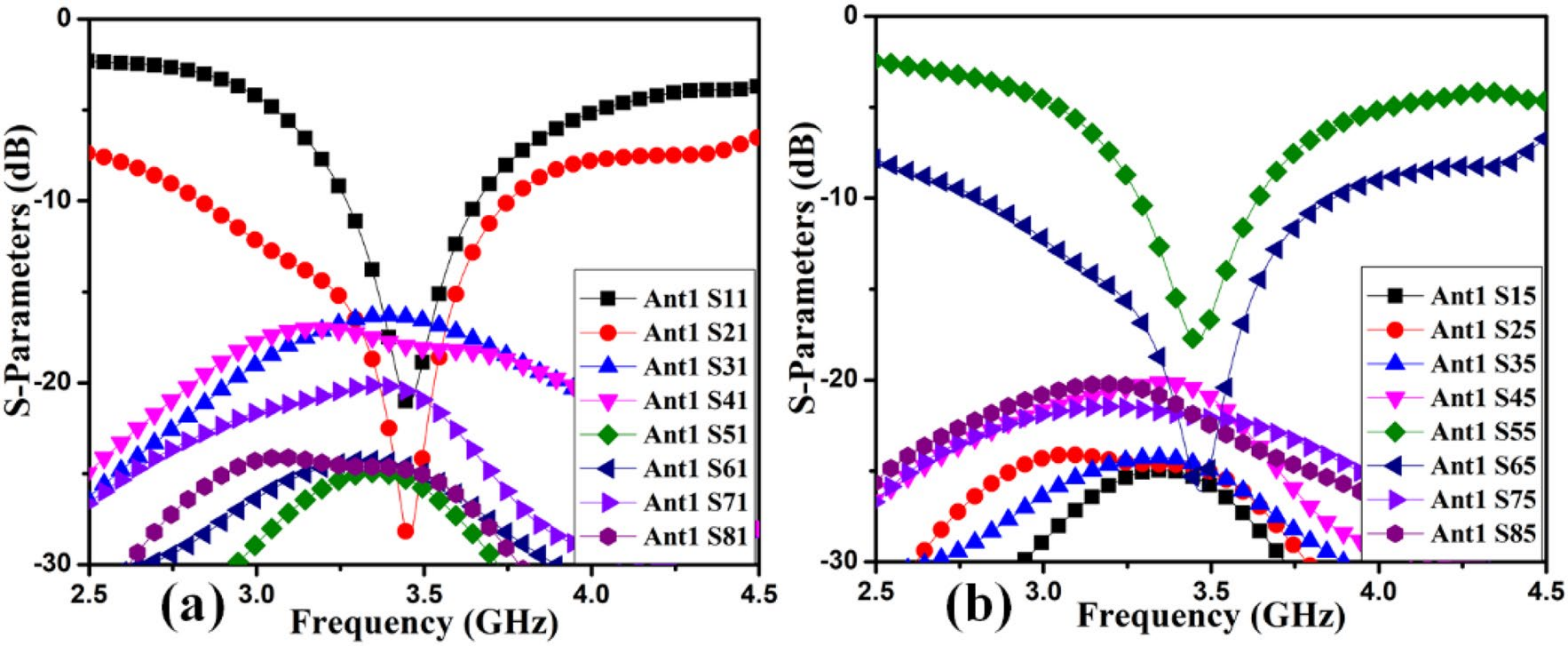
Simulated S-parameter results (**a**) Ant 1, (**b**) Ant 5.

The MIMO antenna comprises dual pairs of similar M-pattern antenna members (Ant 1–4, Ant 5–8), as shown in Figs. 11 and 12. An analogous pair of antennas is first situated at the leftward and rightward edges of frames (Ant 1–4), then at the upper and rear sides of the frame (Ant 5–8). The S-parameters simulated results of the 8-members closely coupled MIMO antenna is produced in Fig. 13. The antenna members Ant1, Ant2, Ant3, and Ant4 are identical. They have similar S-parameter results, as illustrated in Fig. 13(a). Similarly, antenna members Ant5, Ant6, Ant7, and Ant8 are identical and have identical S-parameters, as shown in Fig. 13(b). The M-pattern antenna members are located close to one another, and the gap between antenna members is around *0.0047λ*_*g*_* (0.2 mm)* at *3.45 GHz*. The Ant1 radiates from 3.26–3.6 GHz, and the Ant5 radiates from 3.26–3.63 GHz. The minimum isolation in the projected 8-member MIMO antenna is 17 dB at 3.45 GHz.

**Figure 14:**
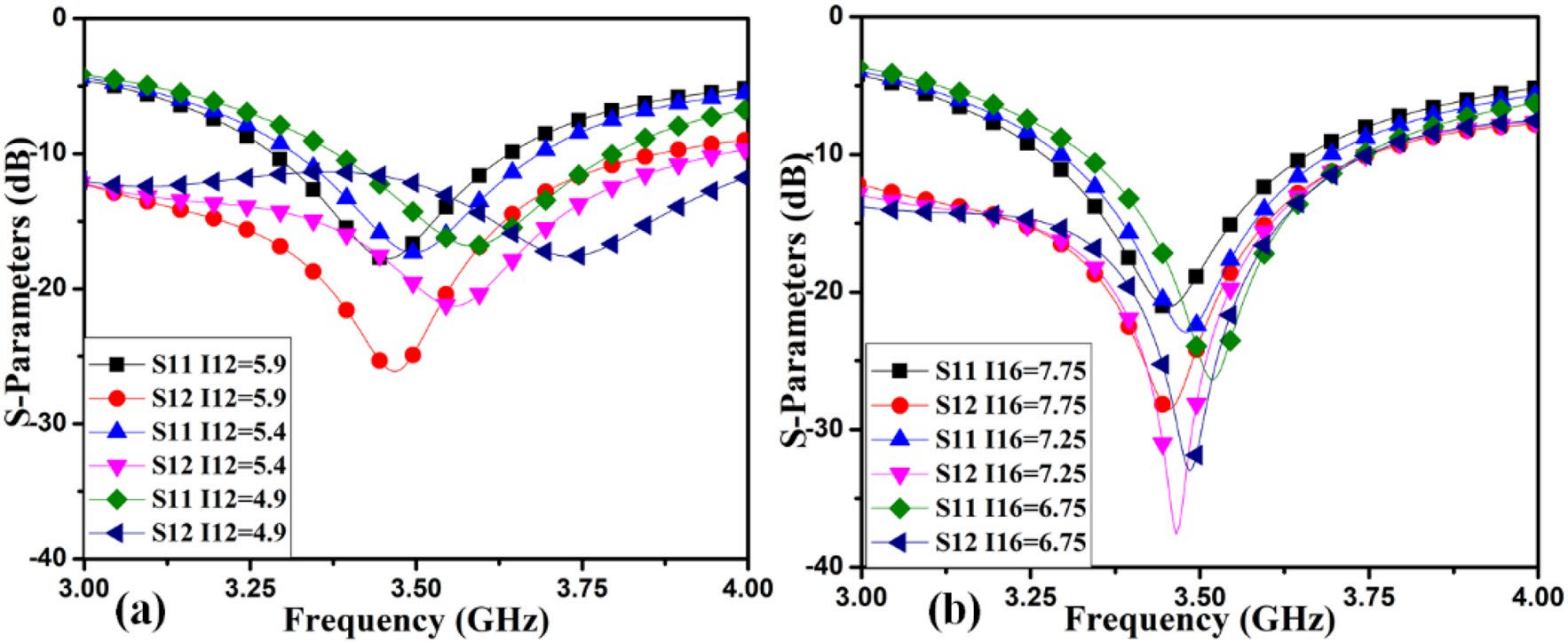
Simulated S-parameter parametric results (**a**) I12 variation, (**b**) I16 variation.

**Figure 15:**
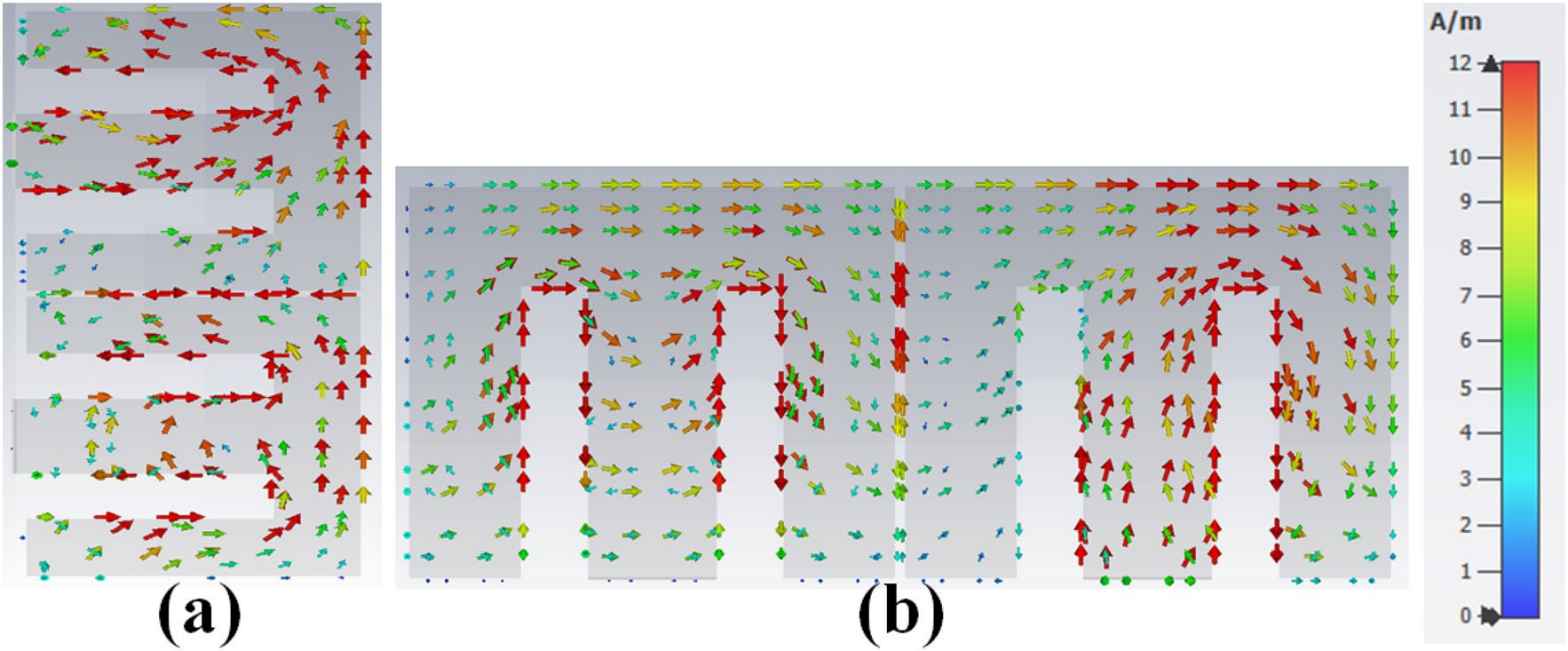
The density of surface current (**a**) Ant 1, (**b**) Ant 5 at 3.45 GHz.

The radiating frequency and isolation of the MIMO (M-shaped) antenna adjust along with the dimension of the M-pattern arm and ground defects; some parametric outcomes associated with the design process are shown in Fig. 14. The closely coupled arms of the MIMO members help improve the structure's isolation by canceling out coupled surface current, as shown in Fig. 15. The optimized measurements of the M-pattern MIMO antenna are shown in Figs. 10, 11, 12. In contrast, entire lengths are presented in Table 2.

## Results and analysis

### 2-member MIMO (M-shaped) antenna

**Figure 16:**
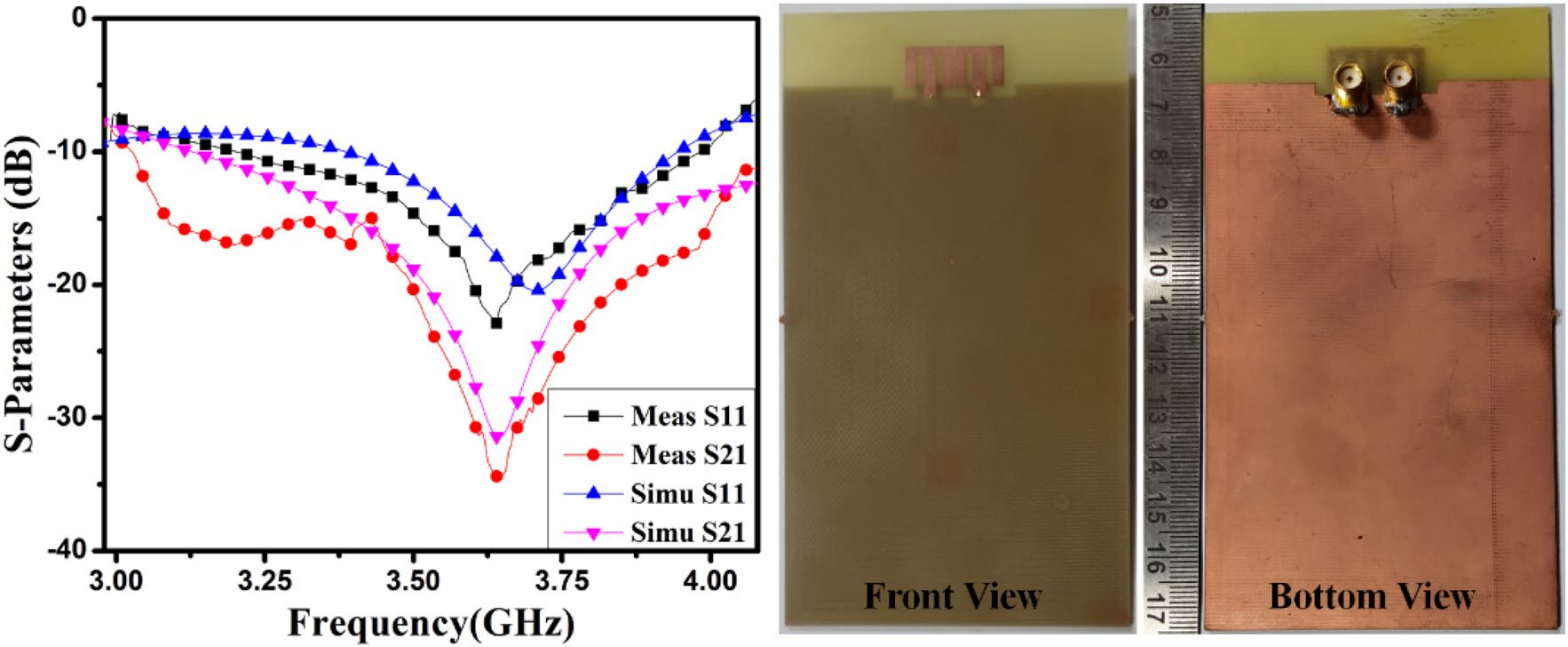
The proposed customized MIMO (M-shaped) antenna measured S-parameters (fabricated sample in enclosure).

The closely-coupled improved MIMO antenna is manufactured with the help of the S63 LPKF Machine, shown in Fig. 16, with the attached S-parameter results. Its features are estimated using a vector network analyzer(VNA) N9916A Keysight Field Fox.

S-parameter results of the optimized closely coupled M-pattern MIMO antenna are matched with the simulated in Fig. 16. The MIMO antenna radiates from 3.2–3.98 GHz, and the isolation between components exceeds 16 dB over the entire frequency range. Some variances are equated to the simulation outcomes in the measurement outcomes expected to manufacture tolerances, soldering errors, and other losses.

**Figure 17:**
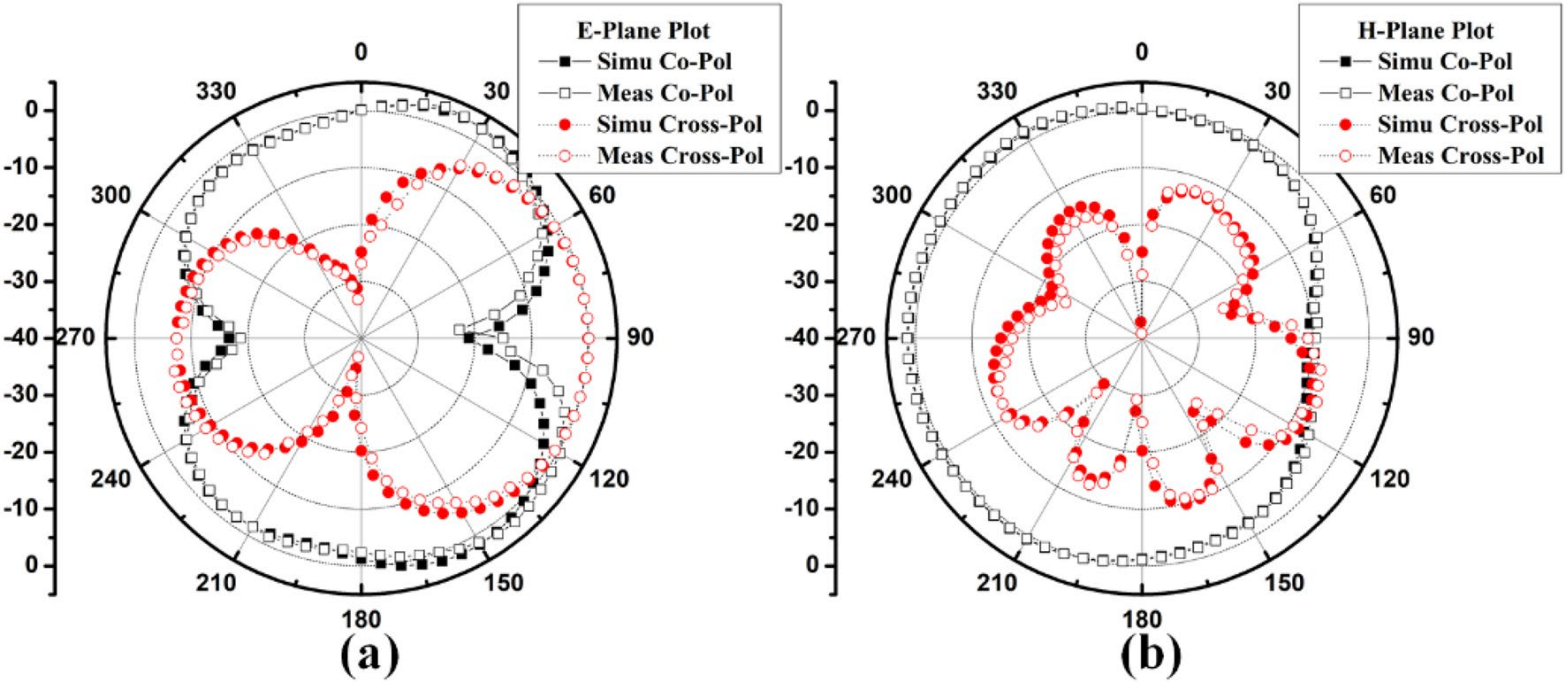
Projected MIMO (M-shaped) antenna polar plot (**a**) E-plane, (**b**) H-plane at 3.65 GHz.

**Figure 18:**
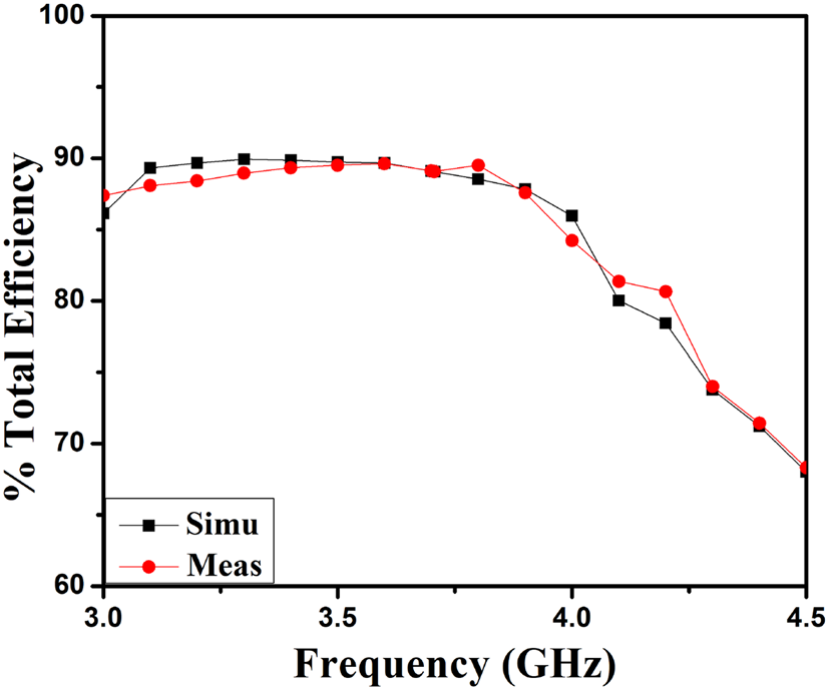
Proposed two members MIMO (M-shaped) antenna total efficiency.

The polar plots of recommended 2-member closely coupled customized M-pattern MIMO antenna are assessed at 3.65 GHz, as demonstrated in Fig. 17 when port one is energized and the second is terminated with a matched load. The polar-plot shapes in H-plane and E-plane have dumbbell-pattern appearances in deteriorated manner. As displayed in Fig. 18, the antenna assessed total efficiencies corresponding nicely with simulated results, and they are advanced to 85% in the preferred band^29^. The assessed outcomes validate that the projected MIMO (M-shaped) antenna system has high efficiency and excellent isolation.

**Figure 19:**
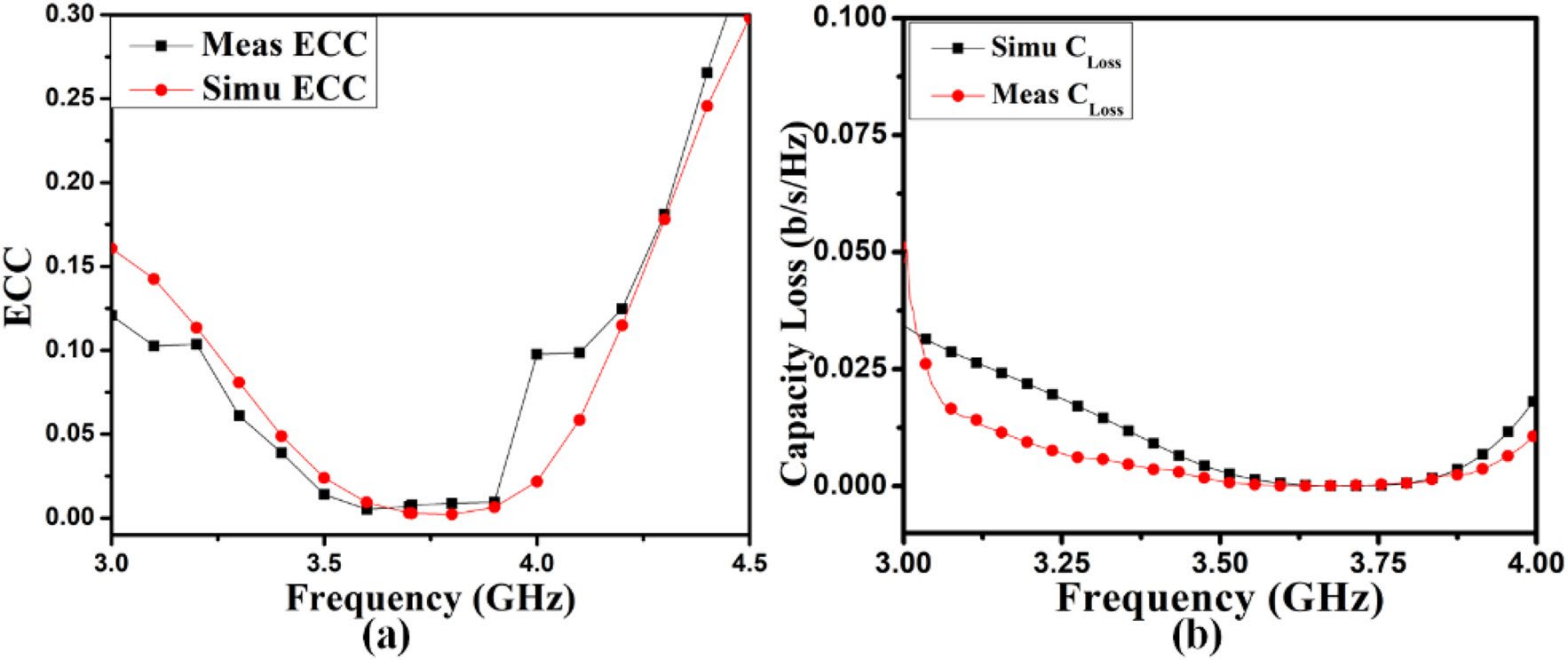
(**a**) ECC and (**b**) C_loss_ results of proposed 2-Members MIMO (M-shaped) antenna.

Consequently, the ECC of the self-isolated 2-member MIMO (M-shaped) antenna is evaluated^30^. The 3D E-fields measured for assessing ECC, as presented in the formula in 2. The projected design ECC is demonstrated in Fig. 19(a). It is below 0.175 in the mandatory frequency range subordinate to the threshold cost of 0.5, as stipulated in the literature^31^.

2 $$ECC = \frac{{\mathop {\left| {\iint\limits_{4\pi } {\left[ {\mathop E\nolimits_{1} \left( {\theta ,\varphi } \right) * \mathop E\nolimits_{1} \left( {\theta ,\varphi } \right)} \right]d\Omega }} \right|}\nolimits^{2} }}{{\iint\limits_{4\pi } {\mathop {\left| {\mathop E\nolimits_{1} \left( {\theta ,\varphi } \right)} \right|}\nolimits^{2} d\Omega \iint\limits_{4\pi } {\mathop {\left| {\mathop E\nolimits_{2} \left( {\theta ,\varphi } \right)} \right|}\nolimits^{2} d\Omega }}}}$$

where, $\overrightarrow{{E}_{1}}\left(\theta ,\phi \right). \overrightarrow{{E}_{2}}\left(\theta ,\phi \right)={E}_{\theta 1}\left(\theta ,\phi \right){E}_{\theta 2}^{*}\left(\theta ,\phi \right)+{E}_{\phi 1}\left(\theta ,\phi \right){E}_{\phi 2}^{*}\left(\theta ,\phi \right)$, $\overrightarrow{{E}_{1}}\left(\theta ,\phi \right)$ is *i*^*th*^ antenna e-fields, when another antenna in the design is fitted with the matched load of 50Ω.

The supplementary important diversity characteristic is the channel capacity loss (*C*_*loss*_). The maximum information rate that may reliably be transmitted through the channel characterizes the channel's capacity, matter-to-channel properties, and the radiation from the antenna. The formula shown as 3 can be used to evaluate the MIMO antenna C_loss_., as explained in^32^. Its estimate is below 0.0125 b/s/Hz, as displayed in Fig. 19(b); this is pretty much under the threshold margin of 0.4 b/s/Hz.

3 $$C_{loss} = - \log_{2} \det (\psi^{R} )$$

where, $$\psi^{R} = \left[ {\begin{array}{*{20}c} {\rho_{11} } \\ {\rho_{21} } \\ \end{array} \left. {\begin{array}{*{20}c} {\rho_{12} } \\ {\rho_{22} } \\ \end{array} } \right]} \right.$$, is the correlation matrix of the antenna at receiving side, that is presented by: $\rho_{ii} = 1 - (\left| {S_{ii} } \right|^{2} + \left| {S_{ij} } \right|^{2} )$, and $\rho_{ij} = - (S_{ii}^{*} S_{ij} + S_{ji}^{*} S_{jj} )$ for *i, j* = *1 or 2*.

### 8-member MIMO (M-shaped) antenna

**Figure 20:**
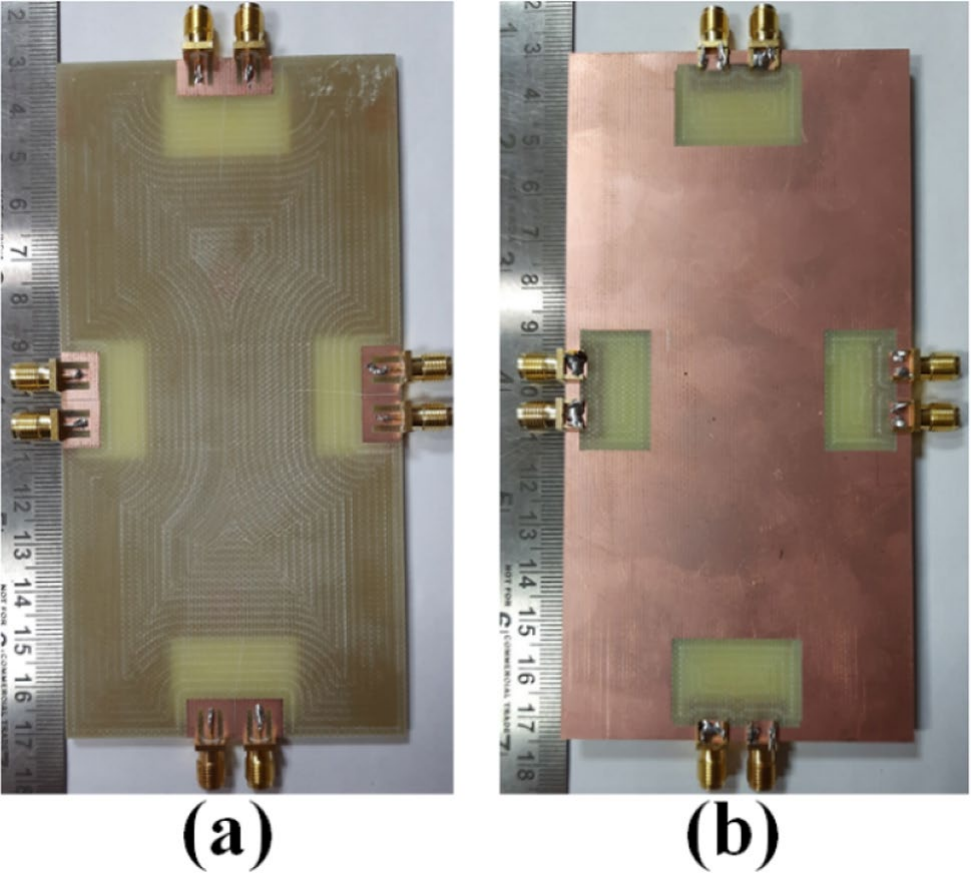
Design sample prototype of 8 Members MIMO (M-shaped) antenna (**a**) Front view, (**b**) Rear view.

**Figure 21:**
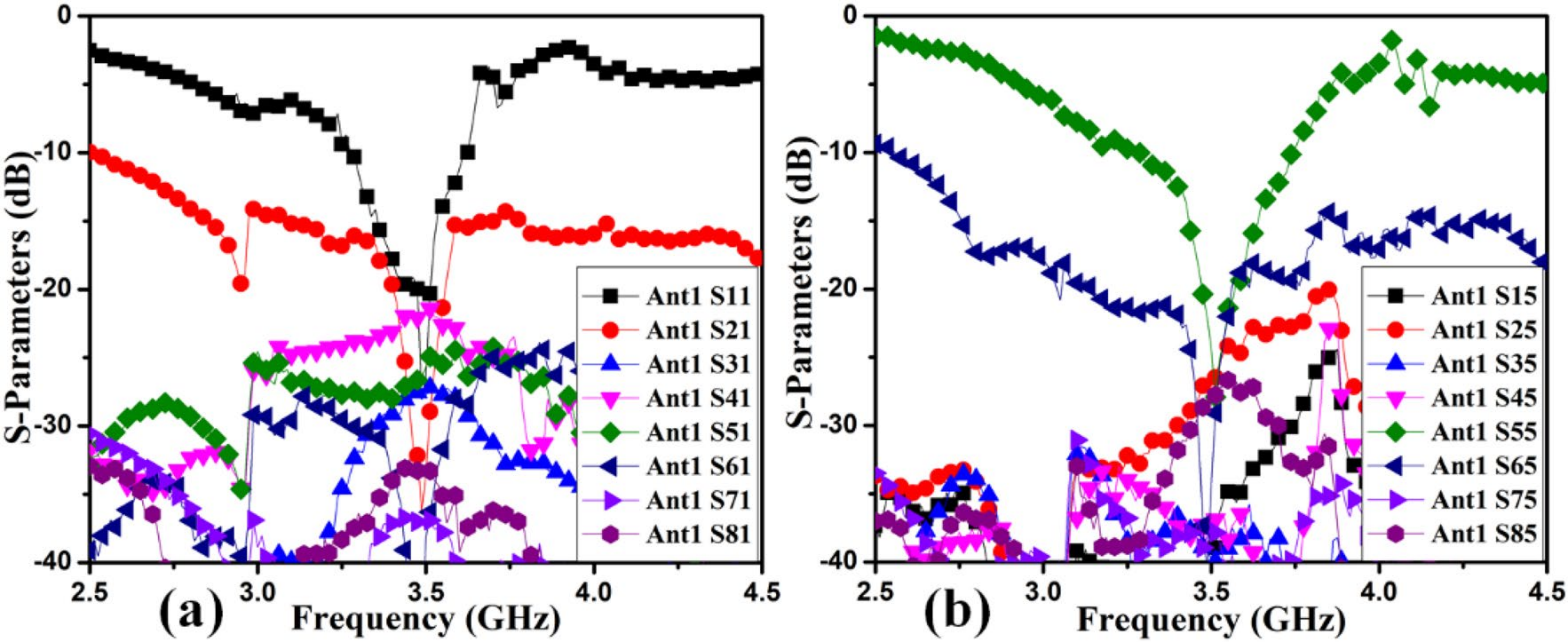
Proposed 8 Members MIMO antenna Measured S-Parameters (**a**) Ant 1, (**b**) Ant 5.

**Figure 22:**
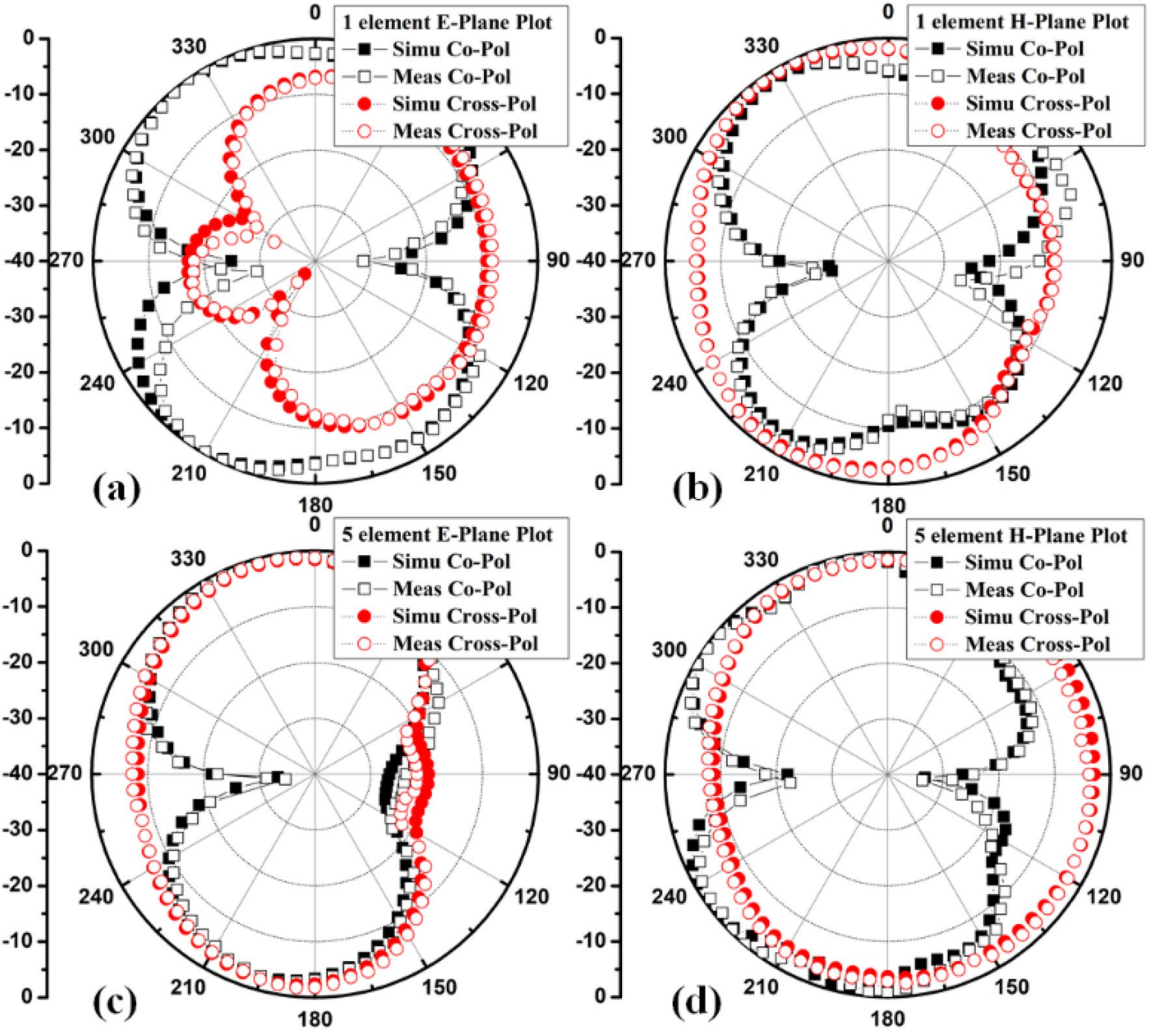
Proposed 8 Members MIMO antenna Polar plot (**a**) Ant 1 E-plane, (**b**) Ant1 H-plane, (**c**) Ant 5 E-plane, (**d**) Ant 5 H-plane.

**Figure 23:**
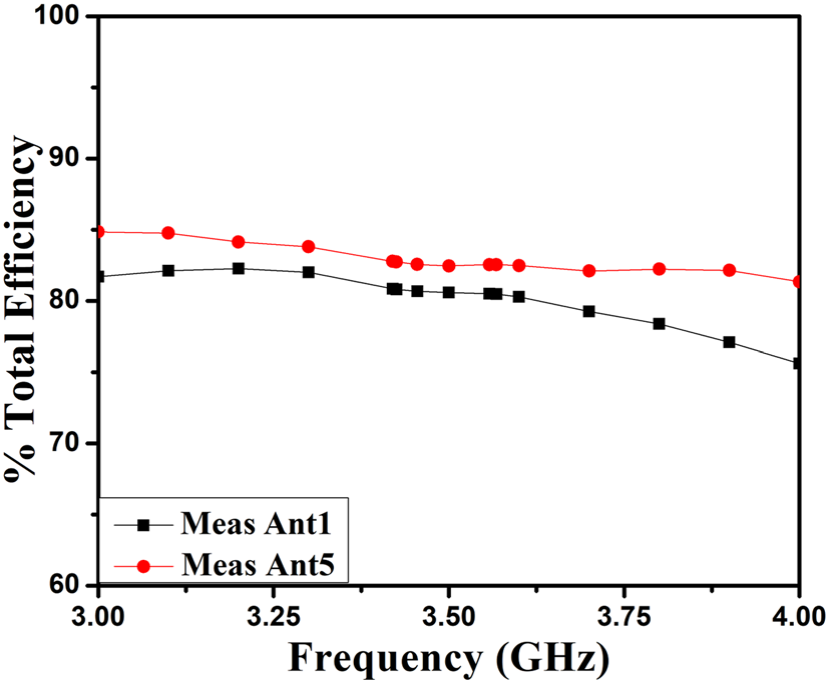
Total efficiency of projected eight members MIMO antenna.

**Figure 24:**
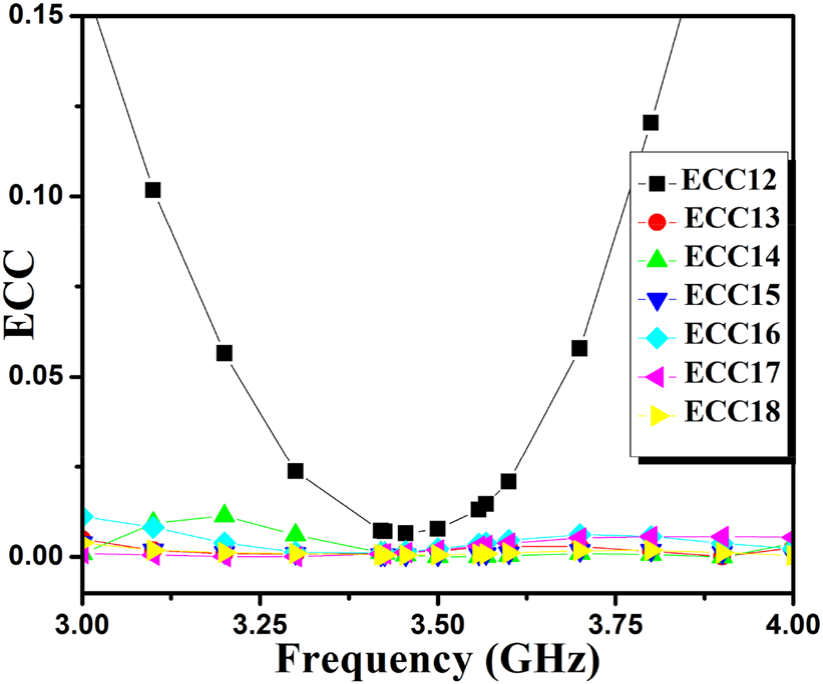
Proposed 8 Members MIMO antenna ECC.

The 8-member tightly coupled MIMO (M-shaped) antenna is manufactured utilizing the LPKF machine, as displayed in Fig. 20. Use N9916A Vector Network Analyzer (VNA) Keysight Field Fox to measure its characteristics. The optimized closely coupled compact self-isolated 8-member MIMO antenna is fabricated to confirm the design results, as displayed in Fig. 20. Its simulated and evaluated S-parameter results are presented in Fig. 21. The MIMO antenna Ant 1 resonates from 3.29–3.66 GHz, and Ant 5 resonates from 3.29–3.74 GHZ with isolation between more than 20 dB members in the desired band. The projected MIMO antenna radiation characteristics are monitored at a 3.5 GHz radiating frequency, as shown in Fig. 22, demonstrating altered omnidirectional qualities. The measured total efficiencies are around 75–85% in the desired bands in Fig. 23. The diversity characteristics of the projected antenna are estimated. The 8-member MIMO antenna ECC is less than 0.02 in the required frequency range, as displayed in Fig. 24.

### Some practical application analysis

#### Mobile frame-based design

**Figure 25:**
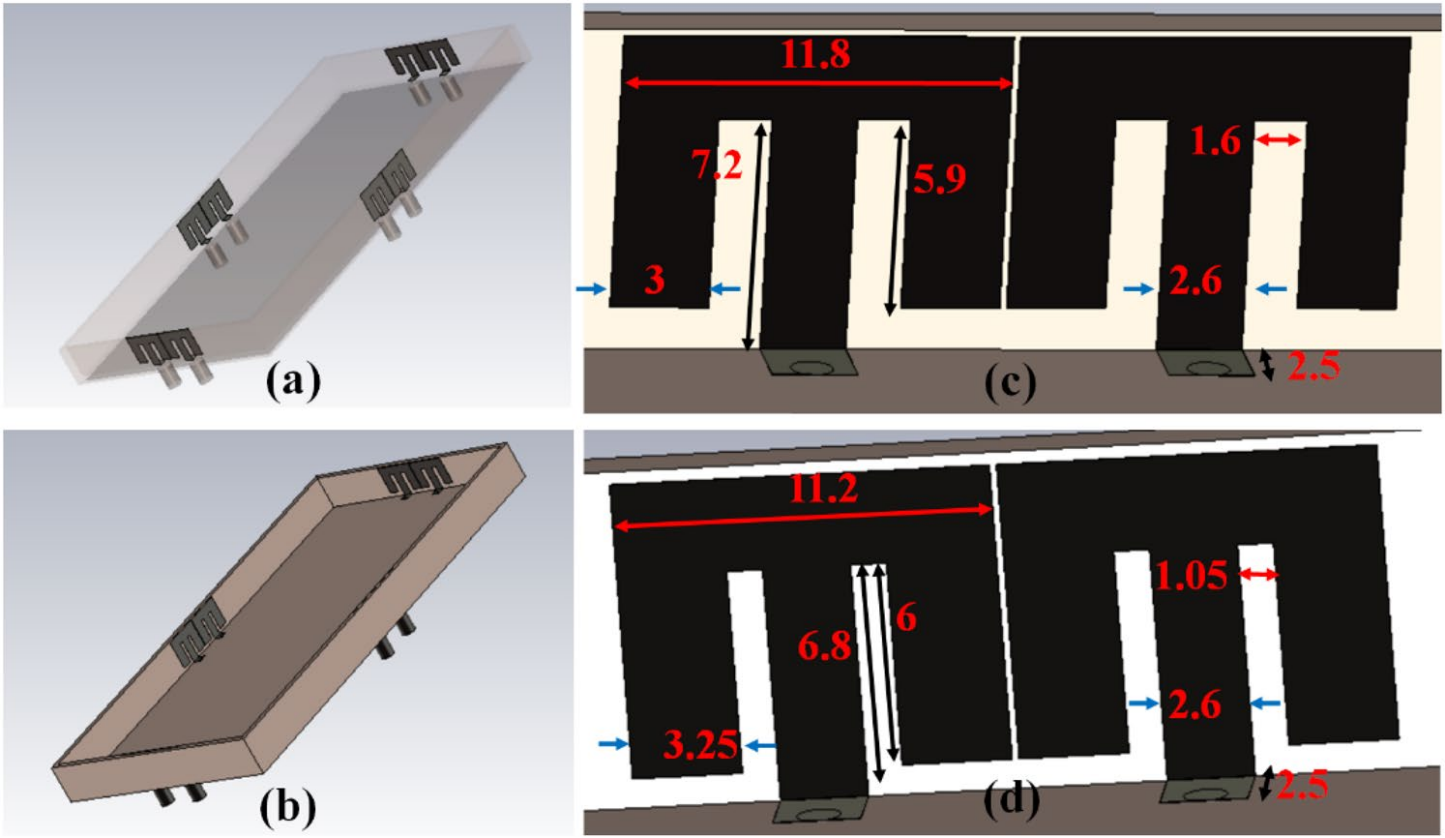
Proposed 8-Members Mobile frame-based MIMO antenna design (**a**) Pairs of eight members, (**b**) structure of mobile frame, (**c**) member one structure and dimension, (**d**) member five detailed structure (all dimensions are in mm).

**Figure 26:**
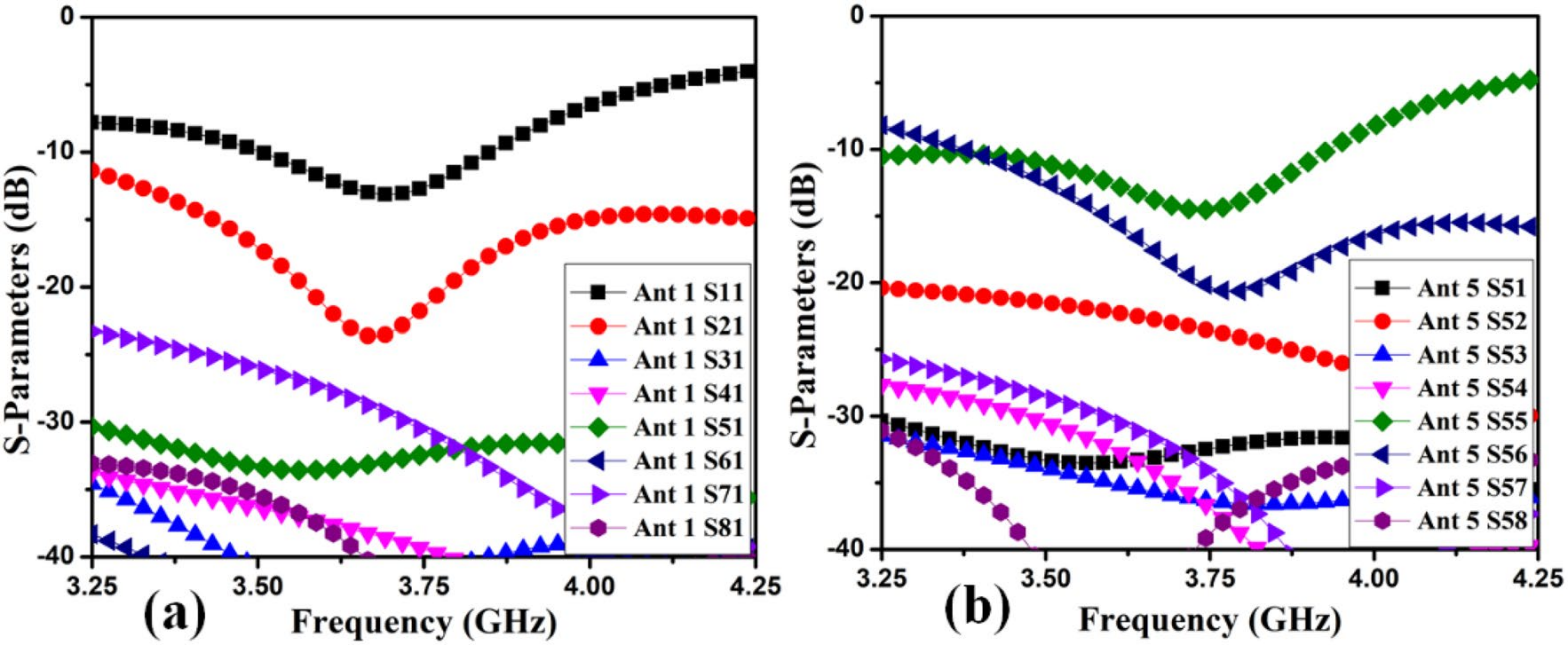
Proposed 8 Members mobile frame-based MIMO antenna S-Parameters simulated results (**a**) Ant 1, (**b**) Ant 5.

**Figure 27:**
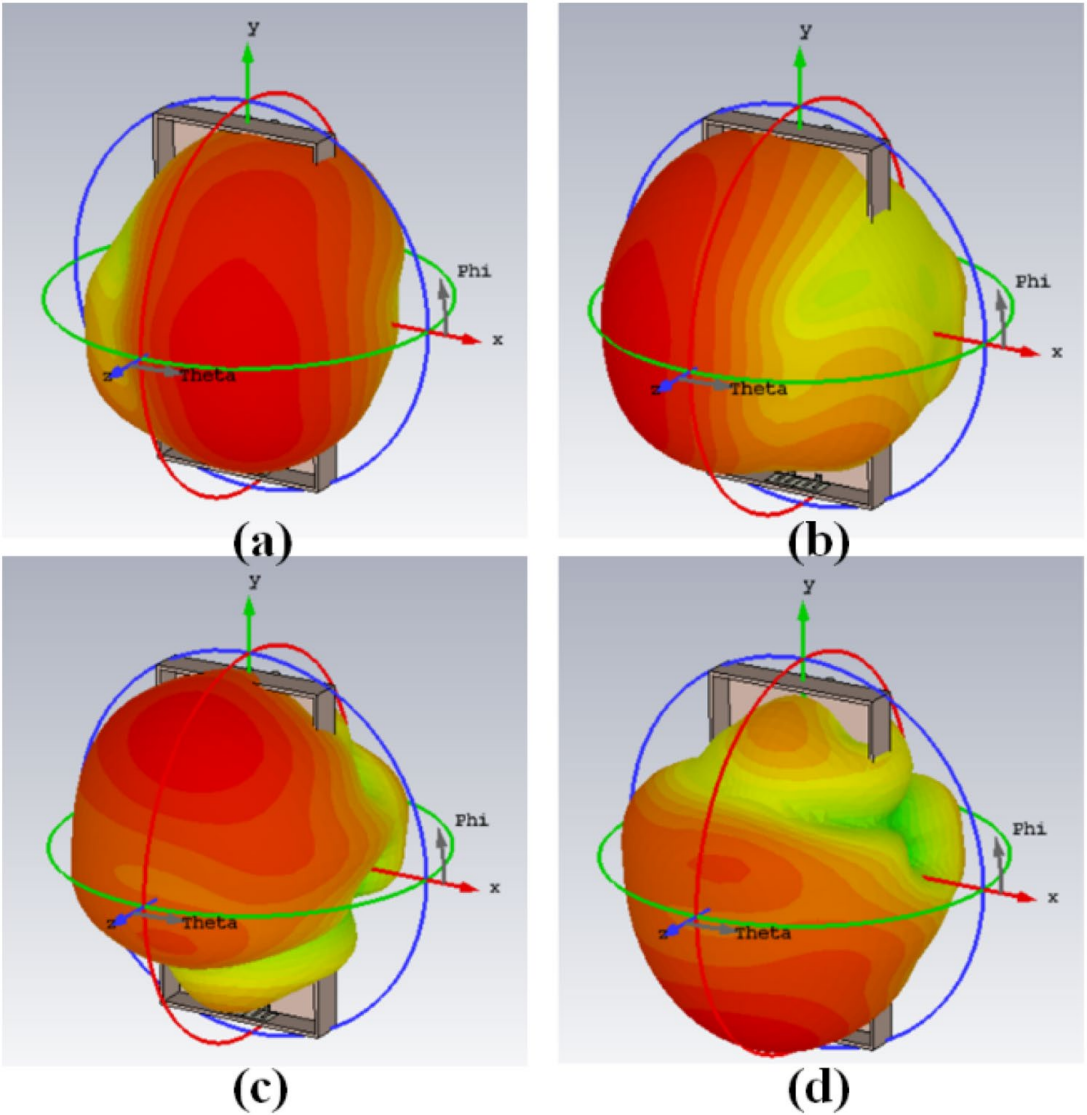
Proposed 8 Members mobile frame-based MIMO antenna 3D radiation patterns simulated results (**a**) Ant 1, (**b**) Ant 2, (**c**) Ant 5, (**d**) Ant 6.

**Figure 28:**
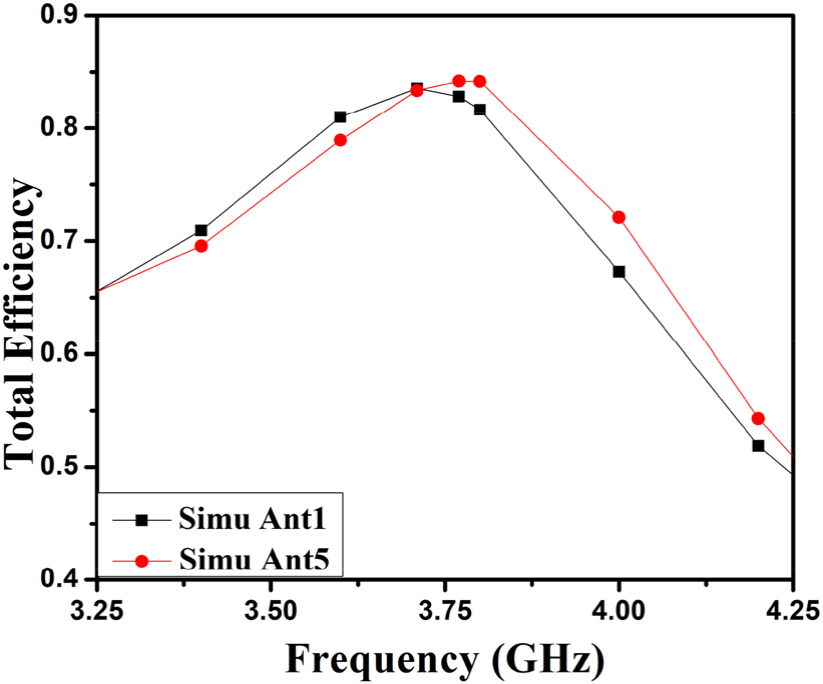
Proposed 8 Members mobile frame-based MIMO antenna total efficiency.

Figure 25 shows the proposed 8-member mobile frame-based MIMO antenna design with detailed geometry^18^. The system dimension is 143.2 mm × 73.2 mm × 1.6 mm, while the ground plane size is 140 mm × 70 mm (with a 1.6 mm clearance along two all sides edges of the ground). The pairs of antenna members are arranged similarly to an 8-member system in mobile frame, with a depth of 1.6 mm and a height of 10 mm. The frame of the antenna considered during design is an FR4 substrate. The S-parameters simulated results of the mobile frame-based MIMO (M-shaped) antenna are presented in Fig. 26 to validate the extension of the proposed idea in higher-order antenna design. The closely coupled antenna member pairs are offered self-isolation properties, and due to orthogonal arrangements, high isolation occurs from other members. The proposed structure covers LTE n48 3.55–3.9 GHz frequency bands. The 3D simulated radiation plot of the MIMO antenna is described in Fig. 27. The total efficiency of antenna members 1 and 5 is presented in Fig. 28. Its maximum efficiency is around 85%, which varies from 70%-85% in the radiating band.

#### Hands effect

**Figure 29:**
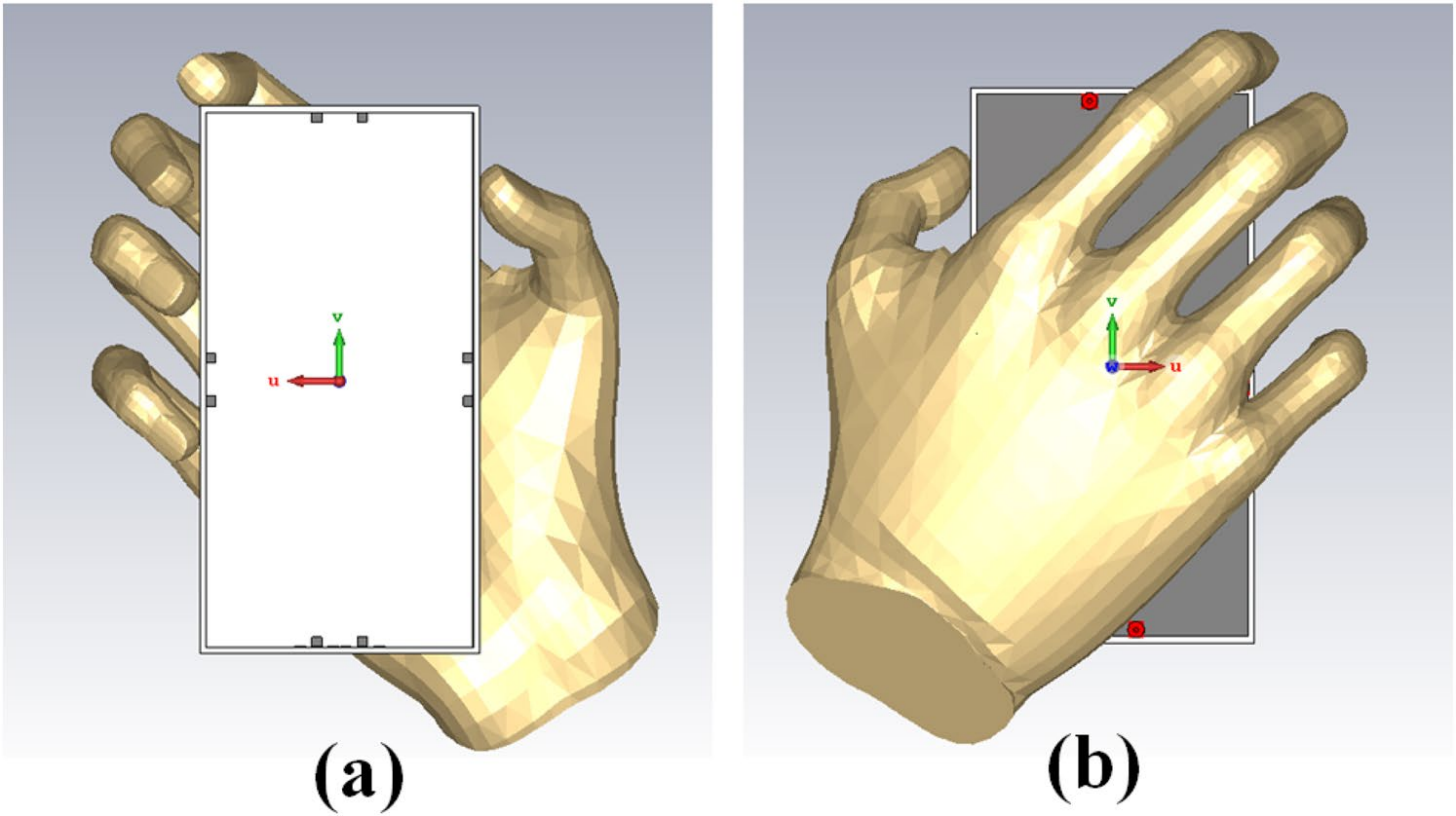
Hand effect on the mobile framed-based MIMO antenna (**a**) front view, (**b**) back view.

**Figure 30:**
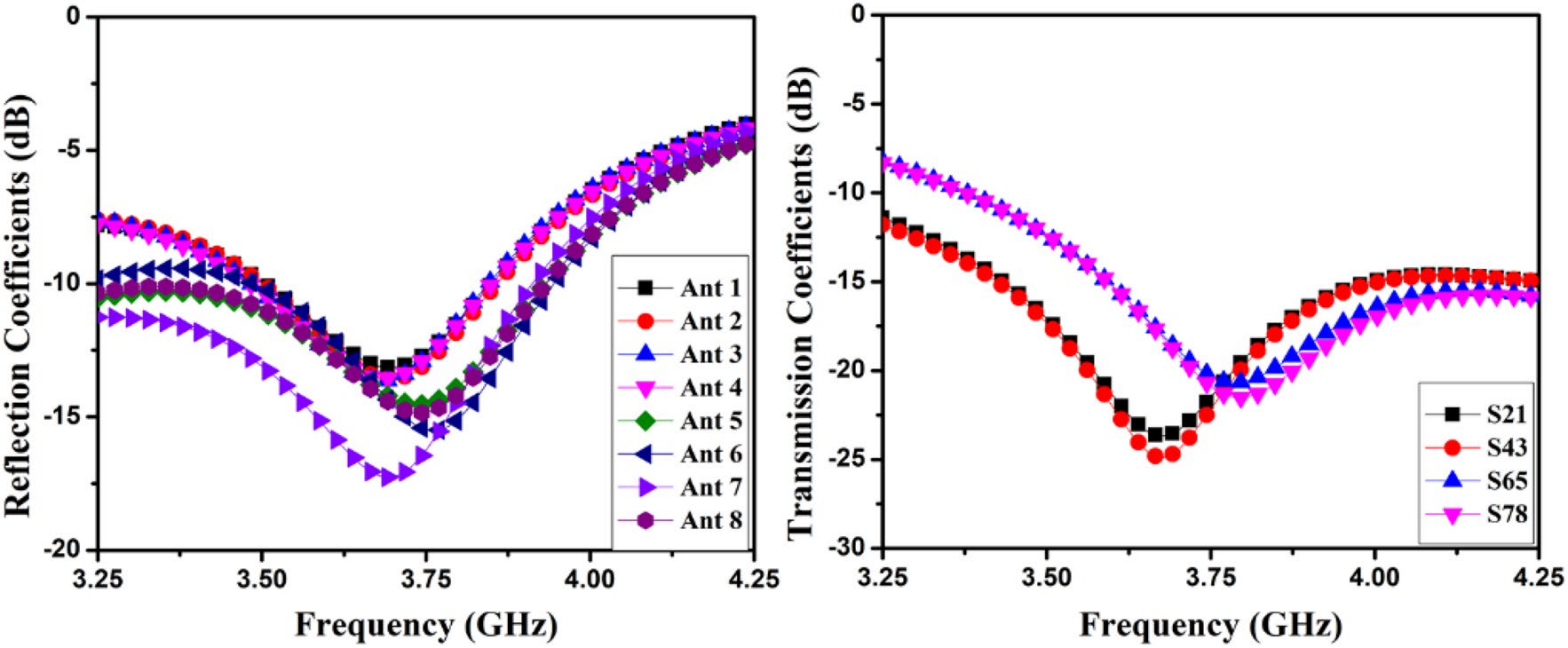
Hand effect on the mobile framed-based MIMO antenna simulated reflection and transmission coefficients.

**Figure 31:**
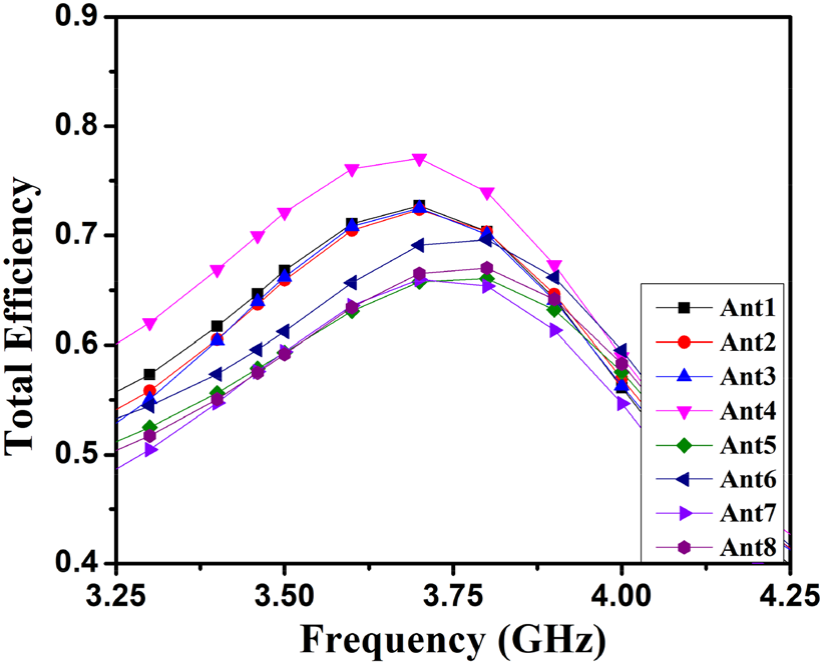
Hand effect on the mobile framed-based MIMO (M-shaped) antenna simulated total efficiency.

The user’s hand effects are analyzed foresight member mobile frame-based MIMO antenna. CST Microwave Studio, the single-handhold arrangement, examines and simulates the proposed antenna. The simulation-based user hands model's front and back views are presented in Fig. 29(a) and (b). According to the results of the hand effects on the S-parameters, the MIMO antenna performed well when the user's hand had high permittivity and high loss characteristics, as shown in Fig. 30. According to Fig. 31, the overall efficiency of MIMO (M-shaped) antenna ranges between 55 and 75% in the current situation.

## Comparison

Above that, the self-isolation antenna design is compared to nearly earlier work in Table 3 from approximately crucial features, including the members' physical linking offerings, bandwidth, minimum isolation,% efficiency, and isolation practices, in the course of a well-understanding of the projected self-isolating impression. Equated with work^6,18,22^, the projected antenna introduces a good reflection coefficient and isolation without a physical link. Other works^17^^,^^21^ utilize the current elimination or reorganization method to enhance the isolation with lesser bandwidth assessed to the projected design. In work^19^, weak field and space diversity methods achieve isolation for limited bandwidth. The asymmetrical arrangement achieves low impedance bandwidth and low isolation with better efficiency^20^. Coupling is terminated by nearby coupling and coupling elimination method for restricted bandwidth^23,24^.

**Table 3:** Comparison with related works.

Refs.	Physical connection	Bandwidth (GHz)	Isolation (dB)	% Effi	Isolation technique
^17^	No	3.4–3.6 4.8–5.0	17 20	> 50	Current redistribution
^18^	Yes	3.3–4.2 (− 6 dB)	10.5	> 63	CM & DM
^19^	No	3.44–3.58	30	–	Poor Field
^20^	No	3.4–3.6 (− 6 dB)	10	> 86	Asymmetrical
^21^	No	3.4–3.6	20	> 38	Cancellation of Current
^22^	Yes	3.4–3.6	20	> 60	Cancellation of Current
^23^	No	3.4–3.6	17	> 58	Coupling Closely
^24^	No	3.3–3.8	20	> 68	Cancellation of Coupling
^25^	No	3.05–3.7 3.2–3.6 & 3.95–4.115	15	> 70	Close Coupled Cancellation using extended structure
^6^	Yes	3.3–7.5 (− 6 dB)	10	> 60	Stub-based Shorting
Prop	No 2-ele 8-ele	3.2–3.98 3.29–3.66	15 20	> 85 > 75	Close Coupled Coupling Cancellation

## Conclusion

In this article, a compact and straightforward self-isolated MIMO (M-shaped) antenna is offered by putting together M-pattern members nearby. It has been established that high isolation is attained due to the closely coupled members canceling out the coupled members' surface currents. The 2-member MIMO antenna radiated a 3.2–3.98 GHz frequency range with 16 dB isolation, and in the frequency range of 3.5–3.82 GHz, more than 20 dB isolation occurred. The diversity characteristics ECC of this antenna are healthier than 0.175. The suggested M-shaped self-isolation MIMO design method can be utilized for a larger array size in MIMO. The 5G application 8-member MIMO antennas are explored in simulation and measurement to validate that. The 8-member MIMO antenna offers 20 dB isolation in the 3.29–3.66 GHz frequency band. The antennas can provide good return loss and isolation with decent diversity characteristics. With the rewards of self-isolation, common ground, wide bandwidth, simple structure, and high efficiency, the projected 5G smartphone arrangement of MIMO antenna design exhibit a promising future.

## Data Availability

All data generated or analyzed during this study are included in this article.
